# Respiratory pathogens and their association with population performance in Montana and Wyoming bighorn sheep populations

**DOI:** 10.1371/journal.pone.0207780

**Published:** 2018-11-26

**Authors:** Carson J. Butler, William H. Edwards, J. Terrill Paterson, Kelly M. Proffitt, Jessica E. Jennings-Gaines, Halcyon J. Killion, Mary E. Wood, Jennifer M. Ramsey, Emily S. Almberg, Sarah R. Dewey, Douglas E. McWhirter, Alyson B. Courtemanch, P. J. White, Jay J. Rotella, Robert A. Garrott

**Affiliations:** 1 Fish and Wildlife Ecology and Management Program, Department of Ecology, Montana State University, Bozeman, Montana, United States of America; 2 Wildlife Health Laboratory, Wyoming Game and Fish Department, Laramie, Wyoming, United States of America; 3 Montana Department of Fish, Wildlife and Parks, Bozeman, Montana, United States of America; 4 Wyoming Game and Fish Department, Laramie, Wyoming, United States of America; 5 Wildlife Health Laboratory, Montana Department of Fish, Wildlife and Parks, Bozeman, Montana, United States of America; 6 Fish and Wildlife Branch, Grand Teton National Park, National Park Service, Moose, Wyoming, United State of America; 7 Wyoming Game and Fish Department, Jackson, Wyoming, United States of America; 8 Yellowstone Center for Resources, Yellowstone National Park, National Park Service, Wyoming, United States of America; Université de Sherbrooke, CANADA

## Abstract

Respiratory disease caused by *Mycoplasma ovipneumoniae* and *Pasteurellaceae* poses a formidable challenge for bighorn sheep (*Ovis canadensis*) conservation. All-age epizootics can cause 10–90% mortality and are typically followed by multiple years of enzootic disease in lambs that hinders post-epizootic recovery of populations. The relative frequencies at which these epizootics are caused by the introduction of novel pathogens or expression of historic pathogens that have become resident in the populations is unknown. Our primary objectives were to determine how commonly the pathogens associated with respiratory disease are hosted by bighorn sheep populations and assess demographic characteristics of populations with respect to the presence of different pathogens. We sampled 22 bighorn sheep populations across Montana and Wyoming, USA for *Mycoplasma ovipneumoniae* and *Pasteurellaceae* and used data from management agencies to characterize the disease history and demographics of these populations. We tested for associations between lamb:ewe ratios and the presence of different respiratory pathogen species. All study populations hosted *Pasteurellaceae* and 17 (77%) hosted *Mycoplasma ovipneumoniae*. Average lamb:ewe ratios for individual populations where both *Mycoplasma ovipneumoniae* and *Pasteurellaceae* were detected ranged from 0.14 to 0.40. However, average lamb:ewe ratios were higher in populations where *Mycoplasma ovipneumoniae* was not detected (0.37, 95% CI: 0.27–0.51) than in populations where it was detected (0.25, 95% CI: 0.21–0.30). These findings suggest that respiratory pathogens are commonly hosted by bighorn sheep populations and often reduce recruitment rates; however ecological factors may interact with the pathogens to determine population-level effects. Elucidation of such factors could provide insights for management approaches that alleviate the effects of respiratory pathogens in bighorn sheep. Nevertheless, minimizing the introduction of novel pathogens from domestic sheep and goats remains imperative to bighorn sheep conservation.

## Introduction

Most large-mammal populations across western North America were decimated following settlement by Europeans, though restoration efforts since the early 20^th^ Century have been particularly successful in reestablishing ungulates in areas where suitable habitat exists [1]. Although management actions have increased bighorn sheep (*Ovis canadensis*) numbers and distribution, the species’ abundance is still less than 10% of historic levels [2]. Currently, most bighorn sheep populations are small and patchily distributed [3], making them more vulnerable to apparent competition [4], predation [5], inbreeding depression [6], and a variety of other threats that collectively amount to the Allee effect [7]. Moreover, respiratory disease has hindered recovery efforts and dictates most bighorn sheep management policies [8].

Respiratory disease epizootics are typically first expressed as all-age mortality events followed by multiple years of enzootic disease that causes high summer mortality of lambs [9]; though the severity and duration of the disease varies. Outcomes following respiratory disease epizootics in bighorn sheep populations range from full recovery to local extinction [10] and long-term recovery is heavily driven by lamb recruitment [11]. Domestic sheep (*Ovis aries*) typically host pathogens that cause this disease in bighorn sheep and experiments commingling domestic sheep with bighorn sheep have collectively resulted in a 98% mortality rate for bighorn sheep [12–14]. Evidence also suggests domestic goats (*Capra aegagrus hircus*) pose a disease risk for bighorn sheep, though the risk appears to be less severe than that posed by domestic sheep [13,15,16]. There is also some evidence that cattle (*Bos taurus*) pose a disease risk [13,17], though cattle are not a natural host for all pathogens associated with respiratory disease in bighorn sheep [18]. Accordingly, disease management efforts have focused on eliminating contact between bighorn sheep and livestock, particularly domestic sheep and goats.

Respiratory disease in bighorn sheep is polymicrobial, with both *Mycoplasma ovipneumoniae* (*M*. *ovipneumoniae*) and *Pasteurellaceae* species contributing to disease [18]. The natural host range of *M*. *ovipneumoniae* is limited to Caprinae species (including domestic sheep and goats) and this bacterium has been associated with respiratory disease epizootics in bighorn sheep [19,20]. Experimental *M*. *ovipneumoniae* inoculation into domestic sheep co-penned with bighorn sheep resulted in epizootic respiratory disease in bighorn sheep, but not domestic sheep [21]. Commingling of bighorn sheep with domestic sheep that had no evidence of exposure to *M*. *ovipneumoniae* resulted in lower mortality of bighorn sheep [22]. *Pasteurellaceae* species including leukotoxigenic *Mannheimia haemolytica (M*. *haemolytica)*, leukotoxigenic *Bibersteinia trehalosi (B*. *trehalosi)*, and *Pasteurella multocida (P*. *multocida)* are also regularly detected in the lungs of bighorn sheep that have succumbed to respiratory disease [23–27], and direct inoculation of leukotoxigenic *M*. *haemolytica* or *B*. *trehalosi* produces fatal pneumonia in bighorn sheep, but not domestic sheep [28]. *P*. *multocida* has been associated with respiratory disease epizootics [20], but not experimentally shown to cause disease.

Rothman’s sufficient-component cause model of disease [29] suggests there are a minimum set of factors and conditionals that cause disease. The history of respiratory disease in bighorn sheep strongly suggests that pathogens originating from domestic livestock are a necessary component of this disease [30] and experimental evidence suggests that introduction of these pathogens to immunologically naïve bighorn sheep is a very strong risk factor, if not sufficient, for epizootics. Other sufficient causes for respiratory disease might require specific ecological conditions along with resident pathogens already circulating in a population [31]. Conversely, enzootic lamb pneumonia in populations that typically follows epizootics could cease or subside due to extinction of pathogens or removal of other component causes related to interactions between the hosts, pathogens, and environment. The identification of pathogen species linked to respiratory disease in bighorn sheep populations without indication of active disease [18] suggests post-epizootic population recovery may result from removal of non-pathogen component causes of the disease. In such populations, variation in host-pathogen interactions (e.g., increases in pathogen virulence, transmission rates, or the accumulation of susceptible individuals) may periodically lead to conditions sufficient for respiratory disease in adults and/or lambs [32]. It follows that characteristics of a population or its environment can influence susceptibility to disease.

Detection error of diagnostic protocols used to detect pathogens, along with a propensity for research to focus on diseased populations, has affected the ability to understand the etiology of respiratory disease in bighorn sheep [19,25,33]. Only recently have molecular and statistical tools to reduce and measure, respectively, detection error of diagnostic protocols been developed and implemented [34–36]. As such, there has been little ability to accurately monitor resident respiratory pathogen communities in bighorn sheep populations. The relative proportion of epizootics involving the introduction of novel pathogens versus resident pathogens is unknown as are other (non-pathogen) causal components of respiratory disease.

The effects of respiratory disease on demographic rates of bighorn sheep, such as recruitment, adult survival, and population growth have been described [37,9,38,11]. However, the relationship between the presence of the pathogens associated with respiratory disease (*Pasteurellaceae* or *M*. *ovipneumoniae*) and population performance has not been well described. Such an assessment could shed light on the relative strength of pathogen versus ecological components as risk factors for respiratory disease. The presence of respiratory pathogens in bighorn sheep populations with strong demographic performance is consistent with ecological components playing a role in causing this disease. Identification of such components could lead to additional disease management strategies that mitigate the effects of resident or novel respiratory pathogens, thereby increasing resilience of bighorn sheep populations to disease.

We assessed bacterial respiratory pathogen communities in a variety of bighorn sheep populations across Montana and Wyoming and related population characteristics and recruitment rates to the presence of *Pasteurellaceae* and *M*. *ovipneumoniae*. The primary objectives of this study were to: 1) assess how common the respiratory pathogens are among bighorn sheep populations; 2) quantify the probability of presence of undetected pathogens by applying recently developed estimates of detection probability for diagnostic protocols used to detect them [36]; 3) assess whether the presence of any specific pathogen or combination of pathogens was associated with population characteristics or recruitment rates; and 4) assess how frequently populations exhibited characteristics indicative of stability and viability in the presence of a given pathogen. Given the long history of domestic sheep grazing in the two states [30], we predicted the respiratory pathogens would be hosted by the majority of study populations. We suspected the effect of respiratory pathogens on demographic performance depended on ecological factors. This combined with our first prediction led us to predict no association between demographic performance of our study populations and presence of respiratory pathogens.

## Methods

### Ethics statement

Capture and handling of animals reported herein comply with scientific guidelines and permits acquired from the State of Montana, the State of Wyoming, and the National Park Service. All animal capture and handling protocols were approved by Institutional Animal Care and Use Committees at Montana State University (Permit # 2011–17, 2014–32), Montana Department of Fish, Wildlife, and Parks (Permit # 2016–005), National Park Service (Permit # NPS 2014.A3), or Wyoming Game and Fish Department (Permit # 854).

### Study populations

We sampled 22 bighorn sheep populations that occupied varying habitat types across Montana and Wyoming and had varying disease and management histories (Table 1). We defined populations to correspond with the survey and reporting methods of their respective wildlife management agencies. Most study populations were geographically separated from other study populations, but populations in the Absaroka Range of Wyoming (n = 6), the Rocky Mountain Front of Montana (n = 2), and the upper Missouri River Breaks of Montana (n = 2) had less geographic separation from neighboring populations. Topography in the ranges of the study populations varies from rugged badlands, with 300 meters of vertical relief to large mountain ranges with over 2,000 meters of vertical relief. Annual precipitation in the ranges varies from 30 cm to over 100 cm. Inhabited ecological regions included northern Rocky Mountains, northern Rocky Mountain Foothills, northern rolling plains, and central Rocky Mountains [39]. Study populations primarily occupied public lands but many also used private lands to varying degrees.

**Table 1:** General attributes of the 22 bighorn sheep populations investigated in this study.

Population	Hunt-Area	Estimated pop. size	All-age epizootics	Origin	Sub-pops^1^	Connectivity level	Migratory	Ecological region^2^
Galton	MT-102	70	None	Native	2	Limited	Yes	N. Rocky Mtn.
Perma-Paradise	MT-124	325	None	Restored	2	Isolated	No	N. Rocky Mtn.
Petty Creek	MT-203	160	None	Restored	2	Isolated	No	N. Rocky Mtn.
Lost Creek	MT-213	100	1991,2010	Restored	2	Limited	Yes	Central Rocky Mtn.
Highlands^3^	MT-340	75	1995	Restored	3	Isolated	No	Central Rocky Mtn.
Sun Canyon^4^	MT-499	150	1925, 1932, 1984, 2010	Native	3	Meta Population	Yes	Central Rocky Mtn./ N. Rocky Mtn. Fthls
Gibson Lake North	MT-423	100	1925, 1932, 1984, 2010	Native	5	Meta Population	Yes	Central Rocky Mtn./ N. Rocky Mtn. Fthls
Fergus	MT-482	545	None	Restored	4	Meta Population	No	N. Rolling Plains
Choteau-Blaine	MT-680	770	None	Restored	10	Meta Population	No	N. Rolling Plains
Middle Missouri Breaks	MT-622	400	None	Restored	3	Isolated	No	N. Rolling Plains
Hilgard^5^	MT-302	280	1997	Augmented-Native	3	Isolated	Yes	Central Rocky Mtn.
Upper Yellowstone	MT-399^5^	320	2012, 2014	Native	8	Meta Population	Yes	Central Rocky Mtn.
Stillwater	MT-500a	75	Pre-1920	Native	2	Limited	Yes	Central Rocky Mtn.
Clark’s Fork	WY-1	600	None	Native	>10	Meta Population	Yes	Central Rocky Mtn.
Trout Peak	WY-2	700	None	Native	>10	Meta Population	Yes	Central Rocky Mtn.
Wapiti Ridge	WY-3	850	None	Native	>10	Meta Population	Yes	Central Rocky Mtn.
Yount’s Peak	WY-4	875	2011–2013	Native	>10	Meta Population	Yes	Central Rocky Mtn.
Franc’s Peak	WY-5	710	2011–2013	Native	>10	Meta Population	Yes	Central Rocky Mtn.
Dubois Badlands	WY-22		None	Native	2	Meta Population	Yes	Central Rocky Mtn.
Targhee	WY-6	80	None	Native	2	Isolated	No	Central Rocky Mtn
Whiskey Mtn.	WY-10	850	1991	Native	>5	Meta Population	Yes	Central Rocky Mtn.
Jackson	WY-7	425	2002, 2012	Native	6	Meta Population	Yes	Central Rocky Mtn.

^1^ Subpopulation defined as a group of animals within a population that shares a distinct winter range that may be composed of multiple social groups.

^2^ Ecological region defined according to USDA Handbook 296 [39]

^3.^ Recent demographic data were not available for the Highlands populations

^4.^ Population was monitored as a single population but inhabits multiple hunting districts.

^5.^ One subpopulation was established from another subpopulation in 2015

Study populations included native populations (*n* = 14), restored populations (*n* = 7), and augmented populations (*n =* 1). Some were part of metapopulations (*n =* 13), while others had limited connectivity (*n =* 3) or were mostly isolated (*n* = 6). Population structure ranged from one subpopulation to over 10 subpopulations. Subpopulations were defined as groups of animals sharing distinct winter ranges that may be composed of multiple social groups. Respiratory disease histories also varied among populations: 11 had no history of confirmed respiratory disease, six had a single all-age epizootic, and five others had multiple all-age epizootics. Estimated abundance in individual populations ranged from 70 to 875 animals (Table 1). Rams were harvested from all study populations with average annual ram harvest ranging from 0.4 to 55. Ewes were harvested from seven study populations (Table A in S1 Appendix) with mean annual ewe harvest from these populations ranging from <1 to 24. Density-reduction translocations had occurred in two study populations and augmentation translocations occurred in two study populations since 2011 (Table A in S1 Appendix).

### Demographic and population characteristic data collection

Routine monitoring data from wildlife management agencies provided the most rigorous and consistently collected data available for most populations. Demographic data used in these analyses were primarily collected by Montana Department of Fish, Wildlife & Parks (FWP) and Wyoming Game & Fish Department (WGFD) personnel as part of annual trend counts and age/sex classifications from 2006 to 2017, typically during January-February or March-April. For one study population (Fergus), we used trend counts conducted during the summer (July-August) and classification counts that were collected during the spring (April-May). Classification and trend counts were conducted exclusively during the summer for one population (Choteau-Blaine), which was excluded from the recruitment analysis. Winter classification counts, but not trend counts, were conducted on a near-annual basis for five study populations (Clark’s Fork, Trout Peak, Wapiti Ridge, Yount’s Peak, Franc’s Peak). The majority of population surveys were conducted from an aerial platform, though surveys for several populations with easily accessible winter ranges were conducted from the ground (Sun Canyon, Gibson Lake North, Hilgard, Lost Creek, and Stillwater). Recruitment data were obtained from classification counts of individuals within a consistent area of core winter range across years. If populations were surveyed multiple times within the same season, the maximum number of animals within each age and sex class observed across surveys was recorded as the counts for that year. Biologists overseeing the study populations were queried to censor poor surveys and obtain information regarding population traits including abundance objectives, 10-year trends, number of subpopulations, connectivity level, as well as history of respiratory disease.

### Indices of demographic performance

We characterized demographic performance of study populations by mean recruitment rates, whether they were at or above abundance objectives, and whether they had a growing or stable population trend. We indexed recruitment rates as the ratio of lambs to ewes (lamb:ewe ratio) counted during winter or spring classification surveys. If mean lamb:ewe ratios of a population were ≥ 0.2, we considered average recruitment of the population satisfactory [40]; population size was satisfactory if it was at or above the management objective; and population trend was satisfactory if growth was stable or increasing. We split data from populations that experienced all-age epizootics within the time-frame recruitment data were collected into pre-epizootic and post-epizootic categories for analysis if pathogen data were collected both before and after epizootics. If pathogen data were not collected before epizootics, we excluded demographic data preceding the die-off from analysis. We omitted the Highlands population from the demographic analyses because recent demographic data were not available.

### Animal capture and sampling

We captured bighorn sheep using chemical immobilization, baited drop nets, or helicopter net-gunning and sampled for presence of *M*. *ovipneumoniae*, leukotoxigenic *M*. *haemolytica or Mannheimia glucosida* (combined as *M*. *haemolytica* as these two species are not reliably differentiated by available diagnostic tests [41]), leukotoxigenic *Mannheimia ruminalis* or *Mannheimia* spp. (combined as *Mannheimia* spp. because the ability to identify *Mannheimia ruminalis* from other species was not available during initial years of data collection), leukotoxigenic *B*. *trehalosi*, *lktA* (a necessary gene for production of leukotoxin A by *Pasteurellaceae*), and *P*. *multocida*. We assessed presence of *Pasteurellaceae* by using a sterile polyester-tipped applicator (Puritan #25–806 1PD, Guilford, Maine, USA) to swab the tonsillar crypts (with the aid of a lighted swab extender and tongue depressor). We also assessed the presence of *Pasteurellaceae* by using a sterile polyester-tipped applicator to swab the nasal cavity in a subset of animals. We assessed presence of *M*. *ovipneumoniae* by using polyester-tipped applicators to swab the nasal cavity. We used ix diagnostic protocols were used to detect *Pasteurellaceae* and we used three to detect *M*. *ovipneumoniae*. We opportunistically incorporated diagnostic testing data for *Pasteurellaceae* and *M*. *ovipneumoniae* from lung samples collected from 15 bighorn sheep that died in an all-age respiratory disease epizootic in the Upper Yellowstone Complex during winter 2014/2015.

#### Diagnostic protocols

We used six different protocols to detect the four *Pasteurellaceae* species of interest, five of which were previously described and evaluated for sensitivity [36]. These five protocols all entailed collecting tonsil swabs but varied by the number of swabs collected, the transport media and corresponding environmental conditions, the diagnostic test (PCR and/or culture), and/or the diagnostic laboratory that was used. The protocol not previously described entailed collecting nasal swabs rather than tonsil swabs, but was otherwise identical to one of the previously described protocols. Several diagnostic protocols that we used to sample 12 animals in December 2017 deviated slightly from described protocols. Complete descriptions of all *Pasteurellaceae* protocols, including those used in December 2017, are found in S2 Appendix.

Additionally, we conducted a confirmatory *Pasteurellaceae lktA* PCR test on a subset of 255 swab samples (from 245 individual animals) submitted to Washington Animal Disease Diagnostic Laboratory (WADDL) for *Pasteurellaceae* culture. The primary streak zone of culture plates containing beta-hemolytic *Pasteurellaceae* isolates were preferentially selected for *lktA* PCR, however random plates were selected for *lktA* PCR if beta-hemolytic isolates were not detected in a sufficient number of samples. The primers and protocol for this PCR were described by Walsh *et al*. 2016 [42]. Our sampling approach did not allow evaluation of detection probability for this test.

We used three previously described and evaluated diagnostic protocols to detect *M*. *ovipneumoniae* [36] from nasal swabs. Complete descriptions of the *M*. *ovipneumoniae* protocols are also found in S2 Appendix.

Lungs collected from pneumonia-killed bighorn sheep in the Upper Yellowstone population were grossly inspected by trained personnel who collected tissue samples and submitted them on dry ice to WADDL for *Pasteurellaceae* culture, *lktA* PCR, and *M*. *ovipneumoniae* PCR. We did not evaluate detection probability for any of the pathogen species in lung tissue samples.

### Updating pasteurellaceae detection probabilities

We updated published estimates of *Pasteurellaceae* detection probability (i.e., sensitivity) [36] by appending testing data from 248 bighorn sheep sampled during November 2016 to March 2017 to the dataset used in the publication. We sampled these supplemental animals for *Pasteurellaceae* using the TSB protocol (samples frozen in tryptic soy broth with glycerol) and the TSB-Nasal protocol (see S2 Appendix for complete description of protocols). Detection probability of the TSB-Nasal protocol had not been previously estimated. We could not update detection probabilities for beta-hemolytic *B*. *trehalosi* because we did not detect this pathogen in the supplemental animals. We estimated detection probability for all protocols using a single-species, single-season occupancy model [43] that allowed detection probability for each pathogen to vary by protocol and prevalence to vary by population and year. To minimize the ad-hoc procedures required to address boundary issues when pathogen prevalence was estimated to be 0 or 1, we used the RMark package [44] in Program R [45] to access Program Mark’s [46] ability to use the sine link to model pathogen prevalence.

### Evaluating freedom from infection

Uncertainty in diagnostic test results is critical information when interpreting results of any pathogen surveillance project. We used a Bayesian hierarchical approach that incorporated uncertainty in detection probability at the sample-level to model presence/absence of undetected pathogens at the population-level (i.e., evaluate freedom from infection [47,48]).We assumed in our modeling approach infection status for each pathogen in the study populations was static throughout the period they were sampled for pathogens unless an all-age epizootic occurred. We justified this assumption based on the relatively short duration of the sampling periods (five years) and the regular persistence of enzootic disease in lambs for multiple years following epizootics [3,11]. We assumed that pathogens were not introduced to populations that did not experience epizootics based on evidence that novel pathogen introduction causes all-age epizootics [21,49] or a sharp decline in recruitment rates [11]. For populations where a specific pathogen was never detected, we estimated the probability the pathogen was truly absent as a function of the pathogen- and protocol-specific detection probabilities (Table C in S2 Appendix), the number of times each protocol was conducted per animal (Tables A and B in S2 Appendix), and the number of animals tested using each protocol (Tables A and B in S2 Appendix). Where multiple protocols were used to detect pathogens in a population, we estimated the overall probability of pathogen absence for that population by grouping sampled animals into cohorts with identical combinations of diagnostic protocols. We modeled probability of pathogen absence assuming no prior knowledge of prevalence or probability of presence (i.e., a uniform prior distribution was used for prevalence and the prior probability of pathogen presence was 0.50). We calculated the probability of pathogen presence as the complement to probability of absence. A complete description of this modeling approach can be found in S2 Appendix. We could not estimate detection probability for several pathogen-protocol combinations, presenting a challenge for estimating probability of pathogen presence when these protocols were employed. We addressed the issue separately depending on the reason the parameter was inestimable (see S2 Appendix for detailed description). We determined that pathogen absence was not reliably assessed in a population when probability of pathogen presence was estimated to be ≥0.10.

### Recruitment and pathogen detection analysis

We included the most recent five years of recruitment data for each study population in the recruitment analysis, with the exception of populations where all-age respiratory epizootics have been documented since 2012. In these cases, we treated the population as independent pre- and post-epizootic populations and only included either “population” in the analysis if we assessed the pathogen community before or after the epizootic, respectively. To minimize the extrapolation of recent pathogen data to historic recruitment rates, we restricted the recruitment dataset to the most recent five years for each population. To explore the possibility that novel influential pathogens were introduced to study populations during the time series, we conducted a piecewise regression analysis [50] for each study population using recruitment data dating back to 2006. We used AIC_c_ model ranking to assess if and when recruitment rates fundamentally changed, indicating a potential change in the pathogen community (S1 Appendix).

We assessed differences in mean recruitment rates with respect to the detection or non-detection of different respiratory pathogens in the study populations using a Poisson rate model with a random-intercept for population in the “lme4” package [51] in Program R. We used the following model for each respiratory pathogen, using a random intercept for study populations and a binary fixed-effect variable indicating whether the pathogen was detected in each population:

$${log(lambs}_{ij})=\log\left({ewes}_{ij}\right)+\beta_{0}+\beta_{1}\cdot {Detected}_{j}+b_{j}+\varepsilon_{ij},$$

where:

*lambs*_*ij*_ is lamb count for population *i* in year *j;*

*ewes*_*ij*_ is the ewe count in population *i* in year *j* (the offset term);

β_0_ is the expected lamb count in populations where the pathogen was not detected;

*Detected*_*j*_ is the variable indicating whether the pathogen was detected in population *j* with a coefficient of β_1_;

*b*_*j*_ is the random intercept of population *j;*

and *ε_ij_* is the residual error term for the expected number of lambs in population *i* in year *j;*

We censored populations from a pathogen’s analysis when that pathogen was not detected and the probability of presence was ≥0.10.

Limited geographic separation of some study populations suggested potential that they could be part of a single population rather than separate populations. To assess consequences of this potential, we conducted a secondary analysis using the same methods described above, but where we treated certain populations in close geographic proximity to each other as a single population. We achieved this by aggregating the adjacent populations’ pathogen sampling and annual demographic data. Populations whose data we aggregated for this secondary analysis included those in the Absaroka Range of Wyoming (WY-1, WY-2, WY-3, WY-4, WY-5, and WY-22) and the Rocky Mountain Front of Montana (MT-499 and MT-423).

## Results

### Capture and sampling effort

We captured and live-sampled a total of 821 individual bighorn sheep (Female: 724, Male: 93 Unknown: 14) from 22 populations in Montana and Wyoming between November and March of each year from 2012–2017. We captured 744 animals once, 60 animals twice, 15 animals three times, and two animals four times. Fifty-seven (57) animals were lambs at time of capture, 72 animals were yearlings, 773 were two years or older and age was not recorded for 15 animals. The number of animals sampled from a single population ranged from six (Yount’s Peak) to 145 (Hilgard).

### Updated detection probability estimates

For two of the three pathogens where detection probability could be updated from previous estimates [36], the estimates for previously reported protocols changed little (Fig A in S2 Appendix); however for *P*. *multocida*, the estimated detection probability for the tonsil swab Port-A-Cul protocol decreased from 0.44 (95% CI: 0.06–0.91) to 0.21 (95% CI: 0.01–0.86), and the estimated detection probability for the tonsil swab TSB protocol decreased from 0.13 (95% CI: 0.08–0.20) to 0.02 (95% CI: 0.01–0.05). The new protocol (TSB-Nasal) provided low detection probability for *M*. *haemolytica* (0.10, 95%CI: 0.03–0.30) and *Mannheimia species* (0, 95%CI: *inestimable*), but provided higher detection probability for *P*. *multocida* compared to other culture-based diagnostic protocols (0.31, 95% CI: 0.20–0.45).

### Pathogen detection

**Fig 1:**
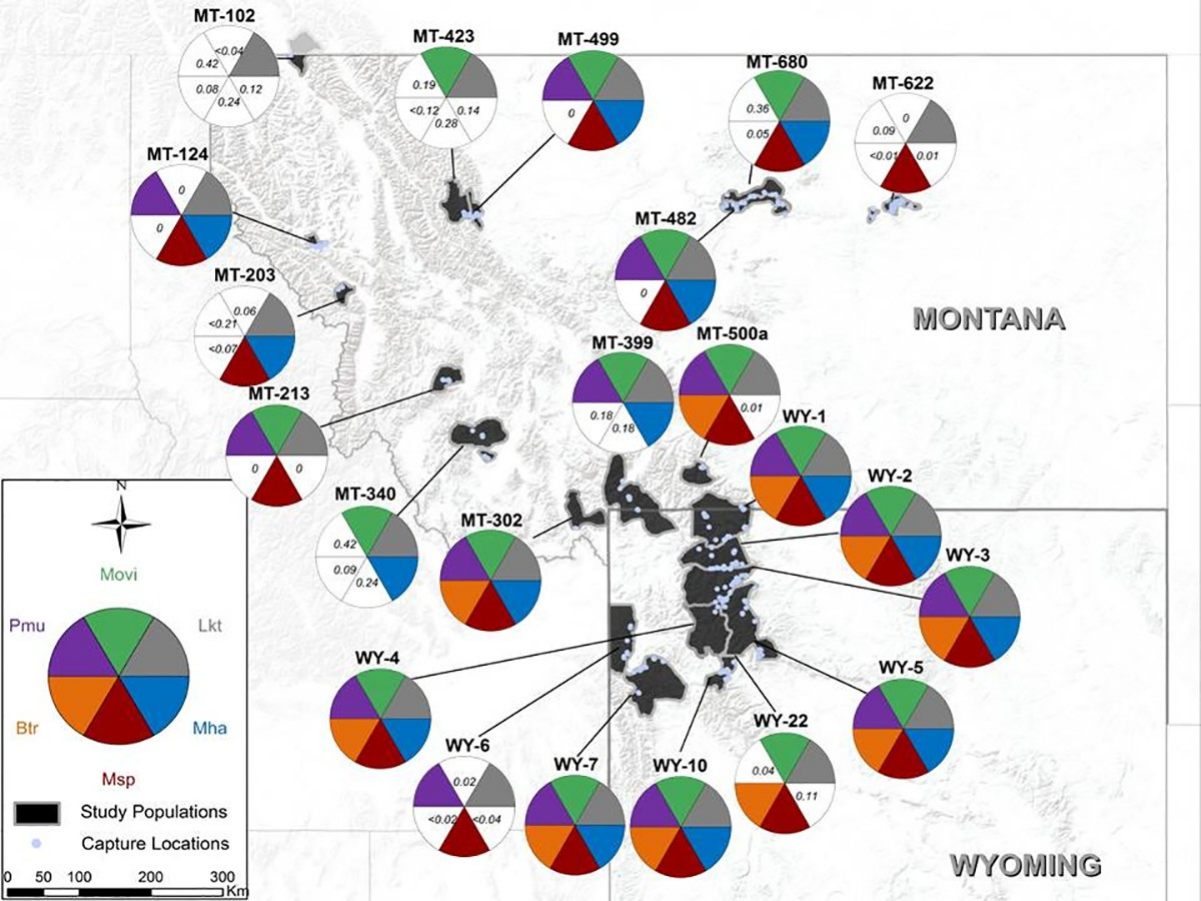
Map of bighorn sheep study populations and detected respiratory pathogen communities. All sections of the pie-charts are fixed to equal size and represent whether the respective pathogens were detected in the study population. The key for pathogen abbreviations are as follows: Movi = Mycoplasma ovipneumoniae, Mha = leukotoxigenic Mannheimia haemolytica/glucosida, Msp = leukotoxigenic Mannheimia spp., Btr = leukotoxigenic Bibersteinia trehalosi, Pmu = Pasteurella multocida, Lkt = Leukotoxin. Where pathogens were not detected, the numbers in the unfilled section indicate the probability the pathogens were present.

The median number of pathogen species(excluding *lktA*) detected per study population was 3.5, and at least one pathogenic agent (including *lktA)* of interest was detected in each study population;, one species was detected in two study populations, two species were detected in two study populations, three species were detected in five study populations, five species were detected in three study populations, and all five species were detected in eight study populations. In two populations, *lktA* was detected without detection of any specific leukotoxigenic *Pasteurellaceae* (Fig 1).

Individually, four of the five pathogenic agents were detected in >65% of the study populations. *M*. *ovipneumoniae* was detected in 17 of 22 (77%) study populations (Fig 1). Leukotoxigenic *M*. *haemolytica* was detected in 15 of 22 (68%) study populations and leukotoxigenic *Mannheimia* spp. was detected in 18 of 22 (82%) study populations. *P*. *multocida* was detected in 15 of 22 (68%) study populations, and leukotoxigenic *B*. *trehalosi* was detected in 10 of 22 (45%) study populations including all but one Wyoming study population and two Montana study populations that are adjacent to Wyoming. *LktA* was detected in all study populations and, therefore, all populations that hosted *M*. *ovipneumoniae* also hosted leukotoxigenic *Pasteurellaceae* (Fig 1). Eighty-eight percent of the 8,460 individual bighorn sheep estimated to exist in the study populations live in populations known to carry *M*. *ovipneumoniae* and leukotoxigenic *Pasteurellaceae*.

Absence of *M*. *haemolytica* was reliably assessed (i.e., estimated probability of presence <0.10) in four of the seven populations (57%) where it was not detected, absence of *P*. *multocida* was reliably assessed in two of the seven populations (29%) where it was not detected, and absence of *B*. *trehalosi* was reliably assessed in 10 of the 12 populations (83%) where it was not detected. Absence of *Mannheimia spp*. was not reliably assessed in any of the three populations where it was not detected. In contrast, absence of *M*. *ovipneumoniae* was reliably assessed in all five populations where it was not detected (Fig 1).

### Population attributes and pathogen detection

Biologists indicated nine of 22 (41%) study populations were below abundance objectives (i.e., estimated population size was less than 90% of objective population size for Montana populations or less than 80% of objective population size for Wyoming populations; Table A in S1 Appendix). Biologists indicated eight of 21 (38%) populations for which adequate demographic data were available had declining 10-year population trends, seven (33%) had stable population trends, and six (29%) had increasing population trends. Of the seven populations thought to have stable trends, five (71%) met herd objectives. Mean five-year lamb:ewe ratios were satisfactory (>0.20) for 16 of 20 (80%) populations where requisite data were available (Table A in S1 Appendix).

Greater than 50% of the study populations had satisfactory recruitment and stable or growing 10-year population trends regardless of whether any specific respiratory pathogen was detected (Table 2). In the five populations where *M*. *ovipneumoniae* was not detected, there was no history of respiratory disease, and four (80%) were determined to be stable or growing. In comparison, 11 of the 17 (65%) populations where *M*. *ovipneumoniae* was detected had a history of respiratory disease, and nine (53%) were considered stable or growing. The populations with *Pasteurellaceae* had similar attributes where between 53% and 66% had a history of respiratory disease, and between 60% and 71% were classified as stable or increasing populations. Characteristics of populations where any specific *Pasteurellaceae* was not detected were similar to populations where the species was detected, one exception being that neither population where *P*. *multocida* was not detected has a history of respiratory disease (Table 2).

**Table 2:** Herd attributes and pathogen detection status for 22 bighorn sheep populations.

	*Mycoplasma ovipneumoniae*		*Mannheimia haemolytica*		*Mannheimia species*		*Bibersteinia trehalosi*		*Pasteurella multocida*	
	+	-^4^	*+*	-^4^	*+*	-^4^	*+*	-^4^	*+*	-^4^
# Populations	17	5	15	4	18	—	10	10	15	2
Previous all-age respiratory disease epizootics	65% (11/17)	0% (0/5)	53% (8/15)	50% (2/4)	44% (8/18)	—	60% (6/10)	30% (3/10)	66% (9/15)	0% (0/2)
Meets abundance objective^1^	53% (9/17)	60% (3/5)	67% (10/15)	50% (2/4)	61% (11/18)	—	60% (6/10)	50% (5/10)	66% (9/15)	50% (1/2)
Adequate recruitment^2^	66% (10/15)	100% (4/4)	93% (13/14)	75% (3/4)	82% (14/17)	—	90% (9/10)	75% (6/8)	87% (13/15)	50% (1/2)
Trend stable or growing^3^	56% (9/16)	75% (3/4)	71% (10/14)	50% (2/4)	59% (10/17)	—	60% (6/10)	66% (6/9)	60% (9/15)	50% (1/2)

^1.^ Meeting abundance objective defined as ≥ 80% of listed population objective for Wyoming populations and ≥ 90% of listed population objective for Montana populations pursuant to state and population-specific management goals. Targhee population objectives are no longer defined according to population size or counts.

^2.^ Adequate recruitment defined as average winter lamb:ewe ratios > 0.20 which has been established as a criteria for healthy populations [40].

^3.^ Based on managing biologists assessment of ten-year population trend.

^4^ Only populations where estimated probability of presence for undetected pathogens was <10% are shown

### Recruitment rates and pathogen presence

The average five-year mean lamb:ewe ratio across the study populations was 0.29, and five-year mean lamb:ewe ratios of individual populations ranged from 0.10 to 0.48. Mean five-year lamb:ewe ratios were between 0.20 and 0.30 for six populations, 0.30 and 0.40 for six populations, and 0.40 and 0.50 for four populations (Table A in S1 Appendix). Mean lamb:ewe ratios were less than 0.20 for four populations. Piecewise regression analysis of 2006–2017 classification data identified break-years for 15 of the 20 (75%) study populations with adequate recruitment data. Six of these 15 (40%) populations experienced an increase in lamb:ewe ratios after the break-year and nine (60%) experienced a decrease. Break-years occurred within the recruitment analysis time-series (after 2012) for three populations (Clark’s Fork, Yount’s Peak, and Whiskey Mountain; Table B in S1 Appendix). *M*. *ovipneumoniae* and leukotoxigenic *Pasteurellaceae* were detected prior to the break-year in two of these populations (Clark’s Fork and Whiskey Mountain) and the other population (Yount’s Peak) was not sampled for pathogens prior to the break-year.

Data from the Upper Yellowstone population were split into pre- (<2013) and a post- epizootic (>2014) categories. Ultimately, 90 population-years of recruitment data (lamb:ewe ratios) from 21 populations were available to assess differences in mean lamb:ewe ratios with respect to the detection of each pathogen species. Censoring data from populations where pathogen absence was not reliably assessed reduced the number of records available for analysis to 73 for *M*. *haemolytica*, 77 for *B*. *trehalosi*, and 77 for *P*. *multocida*. Regression analysis was not conducted for *Mannheimia spp*. because its absence was never reliably assessed; lamb:ewe ratios were summarized for the populations where this pathogen was detected. The frequent co-occurrence of most pathogen species prevented investigation of additive or interactive statistical effects of multiple pathogen species.

**Fig 2:**
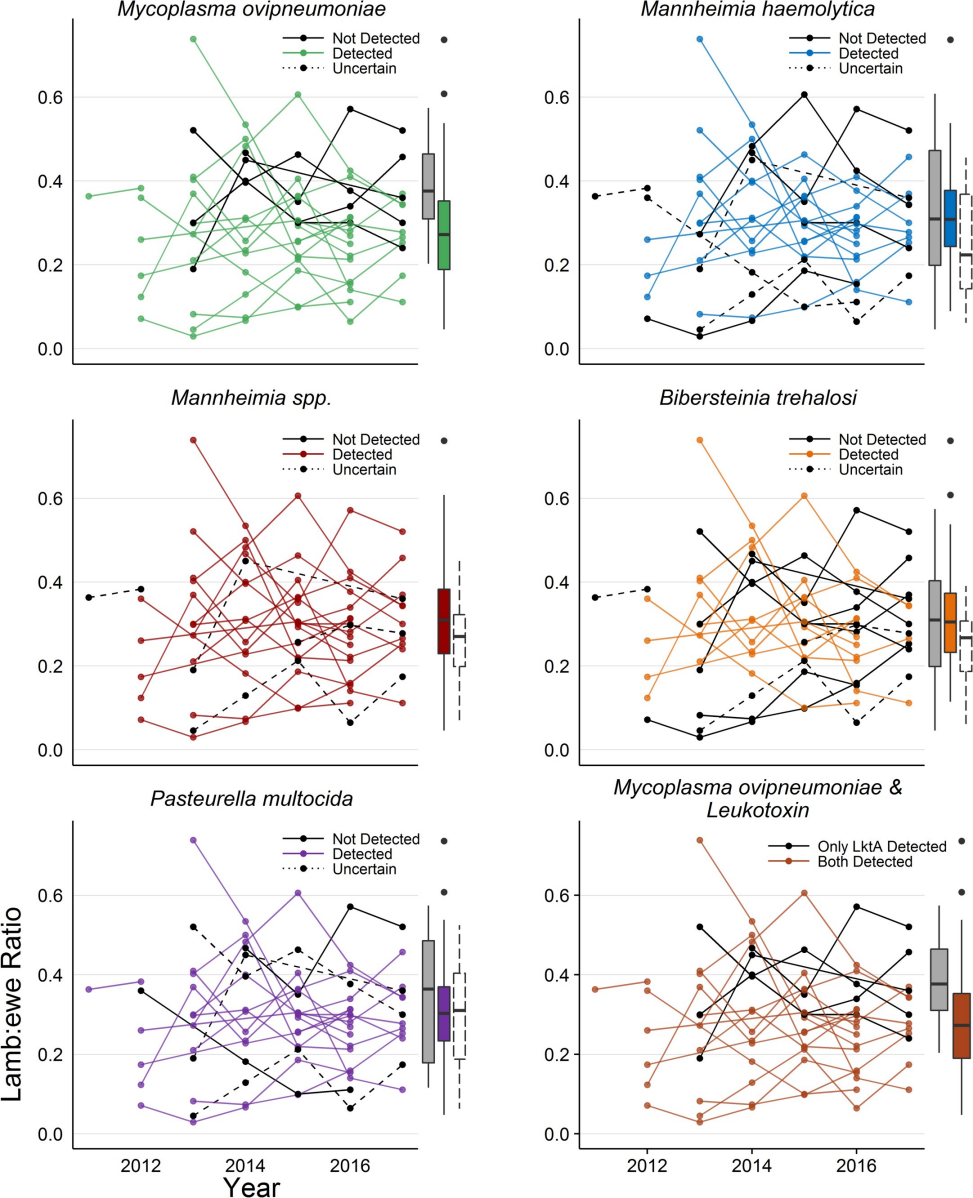
Lamb:ewe ratios and pathogen presence in 21 bighorn sheep populations in Montana and Wyoming. The y-axes show observed lamb:ewe ratios (lambs:100 ewes) and the x-axes show years the data were collected. Grey boxplots show lamb:ewe ratios of populations where the respective pathogen was not detected but probability of pathogen presence was <10%, colored boxplots show where the respective pathogen was detected and unfilled boxplots show where the respective pathogen was not detected and probability of pathogen presence was ≥10%. The boxplot boxes represent the interquartile range (IQR), whiskers represent range of values in the outer quartiles that are within 1.5 times the IQR from the inter quartiles, and points represent values that are not within 1.5 times the IQR from the inter quartiles.

Mean lamb:ewe ratios of individual populations where any specific pathogen was detected ranged from <0.20 to >0.40 (Table 3, Fig 2). For each pathogen species, there were at least four populations that hosted it and had mean lamb:ewe ratios >0.30. There was evidence for an association between detection of *M*. *ovipneumoniae* and lamb:ewe ratios (χ^2^_1_ = 4.49, p-value = 0.03). The estimated mean lamb:ewe ratio was 31% lower (Δ = 0.12) in populations where *M*. *ovipneumoniae* was detected than where it was not detected (Table 3, Fig 2). There was no evidence for an association between detection of any *Pasteurellaceae* species and lamb:ewe ratios (*M*. *haemolytica*: χ^2^_1_ < .01, p-value > 0.99; *B*. *trehalosi*: χ^2^_1_ = 0.83, p-value = 0.77; *P*. *multocida*: χ^2^_1_ = 0.18, p-value = 0.67; Fig 2). Associations between presence of *lktA* and lamb:ewe ratios could not be explored because *lktA* was detected in all study populations. Results from the secondary analysis where neighboring populations were treated as a single populations were consistent with the results of the primary analysis, although evidence for a negative association between lamb:ewe ratios and the presence of *M*. *ovipneumoniae* was weakened and no longer significant at a 0.05 alpha level (χ^2^_1_ = 2.67, p-value = 0.10; Table C in S1 Appendix).

**Table 3:** Model-estimated lamb:ewe ratios and pathogen detection status in 21 bighorn sheep populations.

	*Mycoplasma ovipneumoniae*		*Mannheimia haemolytica*		*Mannheimia species*^*1*^		*Bibersteinia trehalosi*		*Pasteurella multocida*^*1*^	
	*+*	-^2^	*+*	-^2^	*+*	-^2^	*+*	-^2^	*+*	-^2^
# Populations^3^	16	5	15	4	18	—	10	8	15	2
Mean lamb:ewe ratio (95% CI)	0.25 (0.21-0.30)	0.37 (0.27-0.51)	0.29 (0.24-0.35)	0.29 (0.19-0.42)	0.27 (0.22-0.33)	—	0.28 (0.23-0.37)	0.29 (0.21-0.36)	0.27 (0.23-0.33)	0.31 (0.18-0.52)
Minimum mean population-specific lamb:ewe ratio	0.13 (MT-213)	0.31 (WY-6)	0.15 (MT-499)	0.13 (MT-213)	0.12 (MT-213)	—	0.20 (WY-22)	0.13 (MT-213)	0.13 (MT-213)	0.20 (WY-22)
Maximum mean population-specific lamb:ewe ratio	0.41 (MT-302)	0.48 (MT-622)	0.41 (MT-302)	0.48 (MT-622)	0.48 (MT-622)	—	0.41 (MT-302)	0.48 (MT-622)	0.41 (MT-302)	0.48 (MT-622)

^1.^ Absence of *Mannheimia species* was never reliably assessed in populations where they were not detected. Estimates obtained from intercept only model.

^2.^ Data from populations where the probability of presence for a particular pathogen was ≥ 0.10 were not used in the regression analysis

^3.^One population (MT-340) was excluded due to insufficient demographic data

## Discussion

For decades most bighorn sheep populations have been small, isolated, and, consequently, imperiled by a myriad of threats [52,53,3]. Respiratory disease contributes to this predicament by suppressing population growth, range expansion and connectivity among populations. Despite the importance of respiratory disease to the conservation and management of bighorn sheep, its etiology remains unclear due in part to the polymicrobial nature and limited baseline data for pathogen communities hosted by both symptomatic and asymptomatic populations. Integrating what is understood about this disease into management strategies that lead to robust bighorn sheep populations has been challenging. Our findings shed light on approaches that could improve etiological understanding of respiratory disease and develop additional management strategies for bighorn sheep. The negative association between the presence of *M*. *ovipneumoniae* and lamb:ewe ratios along with a lack of disease history in populations where this pathogen was not detected provides additional evidence of its importance and confirm it is an important, perhaps necessary, component of epizootic respiratory disease. There was high variability in the demographic traits of populations hosting both *M*. *ovipneumoniae* and *Pasteurellaceae*; the smallest and largest populations in the study were reservoirs for both pathogen groups and a number of populations hosting the pathogens had average lamb:ewe ratios greater than 0.30. These results suggest that the respiratory pathogens necessary for disease are well established in bighorn sheep populations and the effects of these pathogens might be mediated by ecological factors. Though these findings do not confirm ecological factors influence disease expression, such factors could be significant and may be an important consideration in the management of this species given the challenge of maintaining populations free of respiratory pathogens.

We detected *M*. *ovipneumoniae*, leukotoxigenic *Pasteurellaceae*, and *Pasteurella multocida* in most (13 of 16) populations where the presence of each was reliably assessed (Fig 1). Study populations in the Absaroka Mountains of Wyoming (WY-1, WY-2, WY-3, WY-4, WY-5, and WY-22), the Rocky Mountain Front of Montana (MT-499, MT-423) and the upper Missouri Breaks in Montana (MT-482, MT-680) exist in close proximity to one another and may not have independent pathogen communities due to connectivity. If these populations were aggregated into one population for each region, the number of populations where pathogen presence was reliably assessed would decrease to 12, with the abovementioned pathogens detected in nine of these populations. This proportion is similar to the proportion of bighorn sheep populations across six other U.S. states that were reported to be exposed to *M*. *ovipneumoniae* [14]. Assessment of pathogen communities in additional populations in other regions is needed to confirm whether the common presence of respiratory pathogens in our study populations is representative of most bighorn sheep populations.

The high proportion of study populations already exposed to these pathogen species does not indicate that future pathogen introduction is not a concern. Recent evidence suggests that acquired immunity of bighorn sheep to *M*. *ovipneumoniae* is strain-specific [49]. Exposure to new respiratory pathogen strains introduced from domestic livestock, neighboring bighorn sheep populations, translocated bighorn sheep, and even sympatric mountain goats (*Oreamnos americanus*) [54] could negatively affect any bighorn sheep population. Management agencies face the challenge of simultaneously minimizing the introduction of novel pathogens to bighorn sheep populations while also maintaining population connectivity and viable abundances.

Until recently, respiratory pathogen surveillance efforts had little ability to detect the fastidious pathogens associated with respiratory disease in bighorn sheep [19,25] and current protocols are still imperfect [35,36]. Our findings suggest a number of growing or robust populations that have been used as source populations for translocations may have harbored respiratory pathogens that were subsequently introduced to recipient populations or geographic regions, unbeknownst to wildlife managers. Recent work also suggests that recovery of pneumonic populations is unlikely to be enhanced by introduction of animals naïve to the pathogen strains that those populations carry [49,55]. Thus, a body of evidence suggests that augmenting struggling bighorn sheep populations provides little conservation benefit. The consequence of mixing resident pathogen communities should factor into future decisions to translocate bighorn sheep.

The common detection of *M*. *ovipneumoniae* and *Pasteurellaceae* indicates that resident pathogens are a plausible explanation for some proportion of respiratory disease epizootics. Spontaneous respiratory disease epizootics have been previously reported in captive bighorn sheep [32] and numerous epizootics in free-ranging bighorn sheep have been attributed to a shift to unfavorable ecological conditions that triggered increased virulence or transmission of resident pathogens [56,57]. Epizootics in populations already hosting *Pasteurellaceae* and *M*. *ovipneumoniae* might be caused by introduction of novel pathogen strains or changes in the host, pathogens, or environment that lead to increased virulence or transmission of resident pathogens. Identifying pathogens to the strain level will be necessary to determine which mechanism causes epizootics in such populations. If epizootics are frequently caused by resident pathogens, a broader set of preventative measures are needed to reduce the occurrence of respiratory disease. Our approach to evaluate freedom from infection can be used to establish a framework that quantifies the relative role novel and resident pathogens play in causing epizootics.

Despite the negative association between lamb:ewe ratios and presence of *M*. *ovipneumoniae*, approximately half of the study populations with average lamb:ewe ratios greater than 0.30 hosted *M*. *ovipneumoniae*. This suggests that *M*. *ovipneumoniae* may be necessary for population-limiting respiratory disease in bighorn sheep, but it may not be sufficient. We found no correlation between presence of any *Pasteurellaceae* species we isolated and several indices of population health (i.e., disease history, population trend, achievement of population objective, and recruitment rates). Previous research has also noted little association of *Pasteurellaceae* species with respiratory disease in bighorn sheep populations [18]. However, leukotoxigenic *Pasteurellaceae* have been experimentally shown to cause pneumonia in bighorn sheep but not domestic sheep [12,28,58,59], *Pasteurellaceae* are commonly hosted by healthy domestic sheep [60], bighorn sheep typically develop pneumonia when co-penned with healthy domestic sheep [13], and *Pasteurellaceae* are commonly involved in the pathology of pneumonia in bighorn sheep [17,26,27]. These lines of evidence suggest that *Pasteurellaceae* pose a disease risk to bighorn sheep, particularly in populations where *M*. *ovipneumoniae* is present. Given poor resolution [41] and detection probability [35,36] of diagnostic protocols used to detect *Pasteurellaceae* along with the potential for ecological factors to influence disease expression, these pathogens should not be dismissed as a component cause of respiratory disease based on a lack of correlative evidence.

The demographic variability we observed in populations hosting *Pasteurellaceae* and *M*. *ovipneumoniae* could be explained by undetected differences in pathogen communities. Variation in virulence among pathogen strains that we were unable to distinguish could explain the strong demographic rates of some populations. Differences in virulence could be inherent in the strains or due to attenuation after years of persistence in bighorn sheep populations. Similarly, it is also possible that bacteria were misidentified by the diagnostic tests we used, resulting in false positive errors at the population level. This is most likely to have occurred with *M*. *haemolytica*, which tests often fail to distinguish from *Mannheimia glucosida* [41,42], but false positives could have occurred for other *Pasteurellaceae* or *M*. *ovipneumoniae*. However, high specificity has been reported for the diagnostic tests we used to identify *M*. *ovipneumoniae* and most *Pasteurellaceae* agents [42], suggesting that false positive errors were likely uncommon at the population-level for most pathogens except *M*. *haemolytica*. Although not examined in this study, transmissible sinus tumors have also recently been discovered in bighorn sheep and are associated with increased shedding of *Pasteurellaceae* and *M*. *ovipneumoniae* [61,62]. It is hypothesized that sinus tumors exacerbate upper respiratory tract infections and increase pathogen transmission. Sinus tumors may play an under-recognized role in bighorn sheep respiratory disease and explain some of the variability in demographic rates that we observed among our study populations. Pathogen prevalence in adult females may also explain some variability in recruitment rates but was not assessed in this investigation because it is estimated with poor precision [36]. Lastly, unidentified pathogens could also contribute to disease severity.

If variation in demographic rates of populations hosting both *Pasteurellaceae* and *M*. *ovipneumoniae* is not explained by unobserved differences in pathogen communities or population structure, then the variation of demographic rates, and presumably disease expression, might be mediated by ecological factors. Strong demographic rates of some populations might be temporary states until conditions sufficient for disease expression are met. Certain environments might be less likely to produce conditions sufficient for disease caused by resident pathogens, leading to differential performance among populations with the same respiratory pathogen community and innate immunological potential. Nutrition [63,64], mineral availability [65], stress [56,66,67], migratory behavior [68], and predation [69] have all been shown or hypothesized to influence disease dynamics, sometimes in complex or counterintuitive ways. Given variable population-management histories and over a century of exposure to domestic sheep experienced by many populations, selection may also have increased disease resilience of individuals in certain study populations. Although there are no obvious signs of genetic selection for resistance of bighorn sheep against respiratory disease, it is likely that extensive translocation efforts have interfered with selective processes [1,70]. In populations that have not been augmented it is more likely that selection for disease resistance would have occurred and been maintained.

The scale of our observations could have influenced our results. The larger populations in the study found to host *M*. *ovipneumoniae* and *Pasteurellaceae* are composed of numerous subpopulations which were not all intensively sampled for respiratory pathogens. Our summarization of demographic and pathogen data at the population-level in these cases assumes that each sub-population is exposed to the respiratory pathogens detected across the entire population. Although we cannot validate this assumption, synchronized respiratory disease epizootics across the Sun River metapopulation in Montana (see [71]) suggest it is not unreasonable. Nevertheless, complex population structure of the large study populations could interact with the coarse scale of data collection to mask poor recruitment rates localized in some subpopulations. For example, heterogeneous lamb survival across subpopulations of a bighorn sheep population with enzootic lamb pneumonia has been documented [72]. An emergent property of such asynchrony within populations hosting *M*. *ovipneumoniae* and *Pasteurellaceae* may be the portfolio effect: an ecological phenomenon where asynchronous and volatile components of a system cumulate to a stable system as whole [73]. If this occurs despite the presence of respiratory pathogens in a population, management strategies that increase the structure of populations occupying a particular area could dampen the effects of respiratory disease on bighorn sheep numbers in that area. Such an approach would not diminish the effects of respiratory disease at the subpopulation level, but it may lead to more stability in the number of bighorn sheep occupying a particular area.

There were several limitations of this study not previously discussed. We evaluated freedom from pathogen infection at the population-level assuming there was homogenous mixing of pathogens within populations and that the prior probability of infection was 0.50. These assumptions may not have been met as most populations are comprised of subpopulations and we detected most pathogens in over 50% of the study populations. Violations of these assumptions would have overestimated the probability that undetected pathogens were absent. The estimates of detection probability used in the freedom from infection analysis were calculated assuming perfect specificity; false positive errors could bias these estimates in either direction depending on the underlying cause of the errors. However, most pathogens were detected in most populations and previous studies have determined the diagnostic tests we used have high specificity, indicating that these issues had little potential to affect the overall conclusions of this work. Future applications can build upon our findings to obtain a more informed prior probability of infection. Due to constraints of investigating a large number of populations, our demographic data were limited and we could not collect cause-specific mortality data to assess the proximate drivers of lamb recruitment in the study populations. Survey data collected on a near-annual basis by wildlife management agencies served as the most consistent data source available across the populations. Although longer demographic time-series were available, we limited the data to recent years to maintain the relevance of the demographic data to the pathogen data that we collected. The use of coarse data over a large number of populations limited our ability to investigate the fine-scale processes underlying the patterns we observed. These limitations resulted from a trade-off that allowed for broad inferences at the cost of fine resolution data. Lastly, the non-random selection of study populations limits the extrapolation of these findings, which may not be reflective of other bighorn sheep populations.

### Recommendations and conclusions

This study found that bacterial pathogens associated with respiratory disease are common among the bighorn sheep populations that were studied. Presence of *M*. *ovipneumoniae* in a population increased risk of poor lamb recruitment, likely explained by enzootic lamb pneumonia. However, populations hosting *M*. *ovipneumoniae* and *Pasteurellaceae* were the largest of those surveyed and were often stable or growing. The common presence of respiratory pathogens and the variability among populations that host them confirms plausibility for related hypotheses that: 1) population or environmental characteristics can provide increased resilience in the face of respiratory disease; and 2) respiratory disease epizootics may be caused by either introduction of novel pathogens or increased virulence or transmission of resident pathogens. Although this study lacks the geographic and temporal scale to test these hypotheses, it provides baseline data and recommendations for future studies to test them. These recommendations include:

1. Continue to improve characterization of respiratory pathogen communities by working to adopt diagnostic protocols that provide strong ability to detect pathogens in bighorn sheep populations and identify different strains within pathogen species. Statistical tools should be used to quantify uncertainty in negative test results.
2. Develop a monitoring program for a large and diverse set of bighorn sheep populations where a sufficient number of animals (*n* = 30) [36] can be sampled to assess respiratory pathogen communities. Collect baseline respiratory pathogen data from populations with and without indication of respiratory disease and resample populations following disease epizootics using comparable methods to assess the novel and resident pathogen hypotheses.
3. Characterize attributes of study populations that could influence expression of respiratory disease at the population-level, such as genetics, habitat, nutrition, physiological status, predation, migratory behavior, or population structure.
4. Coordinate and standardize these efforts across states and provinces to collect adequate and comparable data from as many contrasting bighorn sheep populations as possible [74].
5. Continue to maintain and improve upon policies of separation between bighorn sheep and domestic sheep and goats to prevent the transmission of novel pathogens.

We echo recent recommendations for definitions of wildlife health to emphasize resilience and sustainability in the face of numerous anthropogenic and environmental threats rather than the absence of pathogens or disease [75]. Although disease does influence viability, a population that is currently free from disease but is small and isolated with little potential to expand is not necessarily any more viable than one that is currently affected by disease, but has a broad distribution and connectivity with other populations. Our findings suggest that bighorn sheep populations can host a full suite of respiratory pathogens and be healthy under a modern definition of wildlife health. Elucidating the mechanisms driving the patterns we described may aid management of this species.

## Supporting information

S1 Appendix Ancillary demographic summaries and analyses. • **Table A in S1 Appendix. Summary of demographic data for 21 bighorn sheep study populations in Montana and Wyoming that were investigated as part of this study**.
• **Table B in S1 Appendix. Break-years and mean lamb:ewe ratios before and after the break year for each population as estimated by piecewise regression**. Lamb:ewe ratios for each population with requisite data were iteratively split into “before” and “after” groups for every year in the dataset and a Poisson regression model to estimate average lamb:ewe ratios for the “before” and “after” groups was run for each year-based grouping. The break-year was chosen as the year-based grouping with the lowest AIC_C_ score. Break-years were estimated based on before and after recruitment-rates to detect potential years where respiratory pathogens may have been introduced or gone extinct from study populations. Populations whose recruitment rate declined following a break-year are highlighted in red and populations whose recruitment rate increased following a break-year are highlighted in blue. Years of documented all-age respiratory disease epizootics since 2006 are also shown for reference.
• **Table C in S1 Appendix. Comparative results of recruitment and pathogen detection analyses when adjacent study populations were treated as separate populations (primary analysis) vs. aggregated into a single population (secondary analysis).**
(DOCX)

S2 Appendix Ancillary respiratory pathogen sampling and modeling information. • **Table A in S2 Appendix. Animals sampled by population, year, and set of diagnostic protocols that were used to detect *Pasteurellaceae* pathogens.** The numbers under diagnostic protocols indicate the number of times the specified protocol was conducted per individual. Different rows within the same population and year represent “cohorts” of animals that were sampled for respiratory pathogens using the same suite of diagnostic protocols.
• **Table B in S2 Appendix. Animals sampled by population, year, and set of diagnostic protocols that were used to detect *Mycoplasma ovipneumoniae***. The numbers under diagnostic protocols indicate the number of times the specified protocol was conducted per individual. Different rows within the same population and year represent “cohorts” of animals that were sampled for respiratory pathogens using the same suite of diagnostic protocols.
• **Table C in S2 Appendix. Detection probability parameters for *Pasteurellaceae* and *Mycoplasma ovipneumoniae* diagnostic protocols updated and adopted from Butler *et al*.2017.** Bolded beta distributions indicate distributions that were used to model probability of pathogen presence.
• **Fig A in S2 Appendix. Estimated detection probabilities and 95% confidence intervals for five respiratory pathogens in bighorn sheep.** One set of protocols was used to detect the four Pasteurellaceae organisms (shaded) and a separate set was used to detect Mycoplasma ovipneumoniae (not shaded). Detection probabilities for Mannheimia haemolytica, Mannheimia spp., and Bibersteinia trehalosi are for beta hemolytic or leukotoxigenic strains. Protocols that used fee-for-service diagnostic tests are indicated with an asterisk (*) in the legend and above the upper confidence limit. The protocol not previously evaluated is shown in red. The most up to date detection probability estimates are shown with full opacity and previous estimates are dodged left and partially transparent.
• **Fig B in S2 Appendix. Locations of captures and *Mycoplasma ovipneumoniae* detections.** Locations were obtained from coordinates of animal capture locations. Where capture coordinates were not available, coordinates were approximated to match landmarks associated with the capture site.
• **Fig C in S2 Appendix. Locations of captures and leukotoxigenic Bibersteinia trehalosi detections.** Locations were obtained from coordinates of animal capture locations. Where capture coordinates were not available, coordinates were approximated to match landmarks associated with the capture site.
• **Fig D in S2 Appendix. Locations of captures and leukotoxigenic Mannheimia haemolytica detections.** Locations were obtained from coordinates of animal capture locations. Where capture coordinates were not available, coordinates were approximated to match landmarks associated with the capture site.
• **Fig E in S2 Appendix. Locations of captures and leukotoxigenic *Mannheimia species* detections.** Locations were obtained from coordinates of animal capture locations. Where capture coordinates were not available, coordinates were approximated to match landmarks associated with the capture site.
• **Fig F in S2 Appendix. Locations of captures and Pasteurella multocida detections.** Locations were obtained from coordinates of animal capture locations. Where capture coordinates were not available, coordinates were approximated to match landmarks associated with the capture site.
• **Fig G in S2 Appendix. Locations of captures and *Leukotoxin A* detections.** Locations were obtained from coordinates of animal capture locations. Where capture coordinates were not available, coordinates were approximated to match landmarks associated with the capture site.
(DOCX)
