# Supplementary material for: Respiratory pathogens and their association with population performance in Montana and Wyoming bighorn sheep populations
Source: PLoS One. 2018 Nov 26;13(11):e0207780. doi: 10.1371/journal.pone.0207780 (PMC6257920; doi:10.1371/journal.pone.0207780)
Supplement: S1 Appendix — • Table A in S1 Appendix. Summary of demographic data for 21 bighorn sheep study populations in Montana and Wyoming that were investigated as part of this study. • Table B in S1 Appendix. Break-years and mean lamb:ewe ratios before and after the break year for each population as estimated by piecewise regression. Lamb:ewe ratios for each population with requisite data were iteratively split into “before” and “after” groups for every year in the dataset and a Poisson regression model to estimate average lamb:ewe ratios for the “before” and “after” groups was run for each year-based grouping. The break-year was chosen as the year-based grouping with the lowest AICC score. Break-years were estimated based on before and after recruitment-rates to detect potential years where respiratory pathogens may have been introduced or gone extinct from study populations. Populations whose recruitment rate declined following a break-year are highlighted in red and populations whose recruitment rate increased following a break-year are highlighted in blue. Years of documented all-age respiratory disease epizootics since 2006 are also shown for reference. • Table C in S1 Appendix. Comparative results of recruitment and pathogen detection analyses when adjacent study populations were treated as separate populations (primary analysis) vs. aggregated into a single population (secondary analysis). (DOCX) [file pone.0207780.s001.docx]

**S1 Appendix. Ancillary demographic data summaries and analyses**

**Table A.**

| Population | Pop est. | Meets objective^1^ | 10-year  trend | Recruitment survey timing | 5-yr. mean (SD) lamb:ewe Ratio | 5 Yr. Mean (SD) Ewe Harvest | 5 Yr. Mean (SD) Ram Harvest | 5 Yr. Mean (SD) Translocations^2^ |
| --- | --- | --- | --- | --- | --- | --- | --- | --- |
| Galton | 70 | No | Stable | Apr | 0.33 (0.13) | 0 (0) | 1 (0) | 0 (0) |
| Perma-Paradise^3^ | 325 | Yes | Stable^1^ | Apr | 0.36 (0.07) | 3.0 (1.4) | 12.6 (2.8) | 0 (0) |
| Petty Creek | 160 | Yes | Stable | Apr | 0.41 (0.08) | 4.2 (0.8) | 4.4 (3.3) | 0 (0) |
| Lost Creek | 100 | No | Decline | Mar-Apr | 0.10 (0.07) | 0 (0) | 2.0 (1.8) | 0 (0) |
| Highlands | 75 | No | -- | -- | -- | 0 (0) | 0.6 (1) | 2.0 (4.0) |
| Sun Canyon | 150 | No | Decline | April | 0.13 (0.07) | 0.2 (0.5) | 3.8 (1.10) | 0 (0) |
| Gibson Lake North | 100 | No | Decline | April | 0.13 (0.06) | 0 (0) | 2 (0.63) | 0 (0) |
| Fergus | 545 | Yes | Increase | Apr-May | 0.32 (0.05) | 14.6 (7.2) | 16.2 (1.6) | -12 (16.4) |
| Choteau-Blaine | 770 | Yes | Stable | Jul-Aug | -- | 20.4 (4.2) | 23.6 (1.7) | 0 (0) |
| Middle Missouri Breaks | 400 | Yes | Increase | Dec-Feb | 0.48 (0.09) | 24.0 (17.5) | 9.25 (1.3) | 0 (0) |
| Hilgard | 280 | Yes | Increase | Feb-Apr | 0.41 (0.22) | 5.2 (4.3) | 4.2 (1.1) | -14.8 (20.45) |
| Upper Yellowstone | 320 | Yes | Increase | Apr | 0.32 (0.06) | 0 (0) | 3.2 (2.2) | 0 (0) |
| Stillwater | 75 | Yes | Increase | Nov-Jan | 0.43 (0.13) | 0 (0) | 1.2 (0.8) | 0 (0) |
| Clark’s Fork | 600 | Yes | Increase | Jan-Feb | 0.33 (0.14) | 0 (0) | 17.4 (1.3) | 0 (0) |
| Trout Peak | 700 | Yes | Stable | Jan-Feb | 0.27 (0.03) | 0 (0) | 20.0 (2.2) | 0 (0) |
| Wapiti Ridge | 850 | Yes | Decline | Jan-Feb | 0.26 (0.09) | 0 (0) | 34.0 (3.4) | 0 (0) |
| Yount’s Peak | 875 | Yes | Decline | Jan-Feb | 0.26 (0.08) | 0 (0) | 16.0 (3.5) | 0 (0) |
| Franc’s Peak | 710^4^ | No^4^ | Decline | Jan-Feb | 0.26 (0.07) | 0 (0) | 55.0 (20.21) | 0 (0) |
| Dubois Badlands |  |  | Decline | Jan-Feb | 0.19 (0.12) | 0 (0) | 1.5 (1.29) | 0 (0) |
| Targhee^5^ | 80 | No | Decline | Feb-Mar | 0.28 (0.03) | 0 (0) | 0.8 (0.45) | 0 (0) |
| Whiskey Mountain | 850 | No | Stable | Jan-Feb | 0.26 (0.13) | 0 (0) | 14.8 (1.22) | 0 (0) |
| Jackson | 425 | Yes | Stable | Feb | 0.33 (0.07) | 0 (0) | 9.0 (1.82) | 0 (0) |

^1.^ Meeting population size objective defined as ≥ 80% of listed population-size objective for Wyoming populations and ≥ 90% of listed population-size objective for Montana populations pursuant to state and population-specific management goals.

^2.^ Negative sign indicates translocations out of population and a positive sign indicates translocations into population.

^3.^ Population was purposefully reduced through harvest and translocation. Trend considered stable.

^4.^ A single population estimate and objective is established for Franc’s Peak and Dubois Badlands by Wyoming Game and Fish Department.

^5.^ Objective for this population changed to harvest based objective in 2015. This study refers to the previous population-size based objective of 125 animals.

**Piecewise regression to identify break-points in recruitment rates**

A limitation to linking numerous years of recruitment data to respiratory pathogen data only collected during a subset of those years is that the potential exists for pathogens to have been introduced to or gone extinct from the populations within the recruitment data time-series. Therefore, the average recruitment rate of a population could be composed of recruitment data obtained from years where certain respiratory pathogens were present and years where they were not, compromising the ability to accurately characterize recruitment with respect to pathogen presence. We reasoned that the introduction or extinction of any respiratory pathogen that affects lamb survival should result in a pronounced change in recruitment rates of the population after the change, as lamb survival is the vital rate most likely to be affected by respiratory disease [1]. To identify years where an influential respiratory pathogen may have been introduced to or gone extinct from study populations, we conducted a piecewise regression on lamb:ewe ratios for each study population using data collected from 2006-2017. Lamb:ewe ratios from each population were iteratively assigned to one of two groups depending on whether they were observed before or after each year the dataset [2], and the AIC_C_ of the following Poisson regression model was obtained for each year-based grouping, *i*:

${log(\mu}_{j})=\log\left( {ewes}_{j} \right)+\beta_{0}+ \beta_{1}\cdot\left( Year>i \right)$,

where *μ_j_* is the mean lamb count of group *j*. A Poisson rate model was chosen to reflect that the index of recruitment was obtained from count data. The iterative splitting of observations into groups was halted prior to the most recent year of recruitment data to prevent splitting based on a single year’s recruitment rate. The AIC_C ­_scores of these models, as well as that of a single mean model, were ranked and AIC_C_ scores used to determine if the data were best modeled with a single-mean regression line or two-mean regression lines breaking at a specific year within the time series for each population. Although this procedure doesn’t exclude the possibility that introduction or extinction of respiratory pathogens didn’t occur within the resulting datasets, the unobserved occurrence of such events without causing a shift in recruitment rates would suggest whatever changes in pathogen community that occurred did not cause a change in recruitment rates.

**Table B.**

| **Population** | **Documented**  **All-age Epizootics** | **Break year** |  | **Mean lamb:ewe ratio (95% confidence interval)** | |
| --- | --- | --- | --- | --- | --- |
|  |  |  |  | *Before break year* | *After break year* |
| Clark’s Fork | -- | 2014 |  | 0.33 (0.28-0.39) | 0.22 (0.15-0.31) |
| Dubois Badlands | -- | 2010 |  | 0.3 (0.25-0.35) | 0.16 (0.11-0.22) |
| Fergus | -- | None |  | 0.33 (0.27-0.40) | -- |
| Franc’s Peak | 2011-2013 | 2011 |  | 0.32 (0.29-0.34) | 0.24 (0.21-0.27) |
| Galton | -- | None |  | 0.37 (0.29-0.48) | -- |
| Gibson Lake North | 2010 | 2010 |  | 0.30 (0.26-0.35) | 0.10 (0.07-0.14) |
| Hilgard | -- | 2012 |  | 0.24 (0.2-0.3) | 0.41 (0.36-0.47) |
| Jackson | 2012 | 2011 |  | 0.42 (0.38-0.46) | 0.32 (0.29-0.36) |
| Lost Creek | 2010 | 2010 |  | 0.42 (0.37-0.49) | 0.11 (0.07-0.15) |
| Middle Missouri Breaks | -- | 2015 |  | 0.43 (0.39-0.49) | 0.55 (0.46-0.65) |
| Perma-Paradise | -- | None |  | 0.36 (0.33-0.39) | -- |
| Petty Creek | -- | 2009 |  | 0.32 (0.25-0.42) | 0.41 (0.36-0.48) |
| Stillwater | -- | 2013 |  | 0.34 (0.27-0.42) | 0.46 (0.36-0.59) |
| Sun Canyon | 2010 | 2010 |  | 0.28 (0.25-0.33) | 0.10 (0.08-0.13) |
| Targhee | -- | None |  | 0.30 (0.23-0.40) | -- |
| Trout Peak | -- | None |  | 0.28 (0.26-0.31) | -- |
| Wapiti Ridge | -- | 2010 |  | 0.3 (0.28-0.33) | 0.23 (0.21-0.26) |
| Whiskey Mountain | -- | 2015 |  | 0.26 (0.23-0.28) | 0.13 (0.09-0.18) |
| Yount’s Peak | 2011-2013 | 2014 |  | 0.39 (0.34-0.45) | 0.28 (0.23-0.32) |
| Upper Yellowstone | 2012,2014 | 2010 |  | 0.28 (0.25-0.33) | 0.35 (0.32-0.39) |

^1.^ Recruitment data were not analyzed in Choteau-Blaine or Highlands populations due to insufficient winter or spring recruitment data.

**Secondary recruitment and pathogen detection analysis**

Limited geographic separation of some study populations suggested potential that they could be part of a single population rather than separate populations. To assess consequences of this potential, we conducted a secondary recruitment and pathogen detection analysis using the same methods described in the methods section of the main article, but here we treated certain populations in close geographic proximity to each other as a single population. We achieved this by aggregating the adjacent populations’ pathogen sampling and annual demographic data. Populations whose data we aggregated for this secondary analysis included those in the Absaroka Range of Wyoming (WY-1, WY-2, WY-3, WY-4, WY-5, and WY-22) and the Rocky Mountain Front of Montana (MT-499 and MT-423).

**Table C.**

| **Pathogen** | **Populations detected /**  **total populations**^1^ | |  | **∆ lamb:ewe ratio**^2^  **(p:∆ = 0)** | |
| --- | --- | --- | --- | --- | --- |
|  | Primary analysis | Secondary analysis |  | Primary analysis | Secondary analysis |
| *Mycoplasma ovipneumoniae* | 16/21 | 10/15 |  | -0.12  *(0.03)* | -0.10  *(0.10)* |
| *Mannheimia haemolytica* | 13/17 | 9/13 |  | 0.00  *(1.00)* | +0.01  *(0.912)* |
| *Mannheimia species* | 17/17 | 13/13 |  | NA | NA |
| *Bibersteinia trehalosi* | 10/18 | 6/14 |  | +0.01  *(0.77)* | +0.06  *(0.38)* |
| *Pasteurella multocida* | 16/18 | 12 /13 |  | -0.03  *(0.67)* | -0.21  *(0.13)* |

**^1.^** Analyses excluded populations where pathogen was not detected and the estimated probability of it being present in a population was >0.10.

**^2.^** ∆ lamb: ewe ratio equals model-estimated mean lamb:ewe ratio where a pathogen was not detected subtracted from the model-estimated mean lamb:ewe ratio where a pathogen was detected.

**References**

1. Manlove K, Cassirer EF, Cross PC, Plowright RK, Hudson PJ. Disease introduction is associated with a phase transition in bighorn sheep demographics. Ecology. 2016;97: 2593–2602. doi:10.1002/ecy.1520

2. Crawley MJ. Regression. The R Book. John Wiley & Sons, Ltd; 2012. pp. 449–497. Available: http://onlinelibrary.wiley.com/doi/10.1002/9781118448908.ch10/summary
