# Supplementary material for: Respiratory pathogens and their association with population performance in Montana and Wyoming bighorn sheep populations
Source: PLoS One. 2018 Nov 26;13(11):e0207780. doi: 10.1371/journal.pone.0207780 (PMC6257920; doi:10.1371/journal.pone.0207780)
Supplement: S2 Appendix — • Table A in S2 Appendix. Animals sampled by population, year, and set of diagnostic protocols that were used to detect Pasteurellaceae pathogens. The numbers under diagnostic protocols indicate the number of times the specified protocol was conducted per individual. Different rows within the same population and year represent “cohorts” of animals that were sampled for respiratory pathogens using the same suite of diagnostic protocols. • Table B in S2 Appendix. Animals sampled by population, year, and set of diagnostic protocols that were used to detect Mycoplasma ovipneumoniae. The numbers under diagnostic protocols indicate the number of times the specified protocol was conducted per individual. Different rows within the same population and year represent “cohorts” of animals that were sampled for respiratory pathogens using the same suite of diagnostic protocols. • Table C in S2 Appendix. Detection probability parameters for Pasteurellaceae and Mycoplasma ovipneumoniae diagnostic protocols updated and adopted from Butler et al.2017. Bolded beta distributions indicate distributions that were used to model probability of pathogen presence. • Fig A in S2 Appendix. Estimated detection probabilities and 95% confidence intervals for five respiratory pathogens in bighorn sheep. One set of protocols was used to detect the four Pasteurellaceae organisms (shaded) and a separate set was used to detect Mycoplasma ovipneumoniae (not shaded). Detection probabilities for Mannheimia haemolytica, Mannheimia spp., and Bibersteinia trehalosi are for beta hemolytic or leukotoxigenic strains. Protocols that used fee-for-service diagnostic tests are indicated with an asterisk (*) in the legend and above the upper confidence limit. The protocol not previously evaluated is shown in red. The most up to date detection probability estimates are shown with full opacity and previous estimates are dodged left and partially transparent. • Fig B in S2 Appendix. Locations of captures and Mycopla [file pone.0207780.s002.docx]

**S2 Appendix. Ancillary respiratory pathogen sampling and modeling information**

**Pathogen Diagnostic Protocol Descriptions**

***Pasteurellaceae:***

Wyoming*:* Cultures from the tonsils were obtained using sterile polyester applicators (Puritan#25-806 1PD, Guilford, ME, USA) applied to the tonsilar crypts and the outer tonsil surface. Inoculated swabs were immediately used to inoculate one quarter of a Columbia Blood Agar plate (CBA) with 5 % sheep blood (Hardy Diagnostics #A16, Santa Maria, CA, USA). The applicator was then used to resample the tonsil, followed by placement into transport media; Port-A-Cul^TM^ tubes(Becton Dickinson, Franklin Lakes, NJ, USA) or 3ml Amies media without charcoal in a 15 x 103mm culture tube (Triforest Enterprises, Irvine, CA, USA). Samples from the nasal passages were collected using the same type of applicator, but was gently inserted 8-12cm into the nasal cavity while slowly rotating the shaft. The inoculated swab was then placed into transport media as described above. All samples were transported to Wyoming Game and Fish Department Wildlife Health Laboratory (WGFD) and processed within four hours of collection. Bacterial plates were struck to three quadrants using a 1µm loop and incubated at 37^o^C in 5% CO_2_. Tonsil swabs were removed aseptically from the transport media with forceps and used to inoculate one half of a CBA plate. Tissues were aseptically removed from 18oz Whirl-Pak® bags (Nasco, Fort Atkinson, WI, USA), cut to expose an interior area of tissue, then smeared over half of a CBA plate. The plate was struck to two quadrants for isolation and incubated at 37⁰C in 5% CO_2_. Culture plates were read and documented once at ~18-24 hours, and again at ~36-48 hours. Targeted colonies were recultured for isolation and identified using standard biochemical tests [1]. After 48 hours, all bacterial growth on plate was collected with a polyester swab and placed in 15ml Falcon tubes (Corning, Corning, NY USA) filled with sterile phosphate buffered saline (BBL FTA Hemagglutination, Buffer Becton Dickinson, Franklin Lakes, NJ, USA), and vortexed to suspended bacteria. A 250µl aliquot was removed and placed into a PCR tube (PCR clean 1.5 mL safe-lock tubes Eppendorf, Hauppauge, NY USA) for DNA extraction (E.Z.N.A. Tissue DNA kit, Omega Bio-Tek, Norcross, Georgia, USA) per manufacturer’s instructions. Each sample was screened with PCR for the leukotoxin (*lktA*) gene with primers that amplified *lktA* in both *Mannheimia* species and *B. trehalosi* [2]. Positive samples were then analyzed using only the *Mannheimia lktA* gene PCR [3]. Samples positive on the initial PCR and negative on the second were categorized as *lktA* positive *B. trehalosi*. *Mannheimia* *spp* *lktA* assay will amplify *lktA* in *M. haemolytica, M. glucosida*, and *M.ruminalis*. *Mannheimia haemolytica /glucosdia lktA* specific PCR [4] was then performed on those samples positive for *Mannheimia* *lktA*.

TSB*.*A single tonsil swab was collected from animals as described in the Wyoming protocol and placed immediately into a vial of tryptic soy broth with 15% glycerol (TSB; Hardy Diagnostics, Santa Maria, California, USA). Samples were frozen as soon as possible and shipped overnight on dry ice to Washington Animal Disease Diagnostic Laboratory (WADDL) for *Pasteurellaceae* culture following the lab’s standard operating procedures. Swabs remained frozen at WADDL until they were plated by diagnosticians.

TSB-Nasal*.* Samples from the nasal passages were obtained by inserting sterile polyester applicators (Puritan#25-806 1PD, Guilford, ME, USA) 8-12 cm into the nasal cavity and slowly rotating the shaft. The inoculated swab was then placed into immediately into a vial of tryptic soy broth (TSB). Samples were frozen as soon as possible and shipped overnight on dry ice to Washington Animal Disease Diagnostic Laboratory (WADDL) for *Pasteurellaceae* culture following the lab’s standard operating procedures. Swabs remained frozen at WADDL until they were plated by diagnosticians.

Port-A-Cul*.* A single tonsil swab was collected as described, placed in a Port-A-Cul ™ tube and kept chilled until received by the diagnostic lab. Samples were shipped overnight on ice packs to WADDL for *Pasteurellaceae* culture following the lab’s standard operating procedures. Samples were shipped to WADDL as soon as possible, arriving within 72 hours of collection. Samples were plated by WADDL immediately upon receipt.

Plated Culture*.* A single tonsil swab was collected as described and immediately used to inoculate a Columbia Blood Agar (CBA) culture plate with 5% sheep blood (Hardy Diagnostics, Santa Maria, California, USA) and a treated as described in the Wyoming PCR protocol. Following the Wyoming PCR protocol, the plate was struck to three quadrants for bacterial colony isolation the day of sample collection. After ~24 hours, a strip of the primary streak zone was swabbed with a sterile polyester tipped swab, as were any phenotypically distinct colonies present on the plate. This swab was placed immediately into a vial of TSB. Samples were frozen immediately and shipped overnight on dry ice to WADDL for *Pasteurellaceae* culture. Swabs remained frozen at WADDL until they were plated by diagnosticians.

Plated PCR*.* Following completion of the MSU protocol, CBA plates were incubated an additional ~24 hours before bacterial growth was cleared from the CBA plate as described in the Wyoming PCR protocol. Samples were stored at approximately - 20° C until being assessed by the Wyoming Game & Fish Department Wildlife Health Laboratory using the PCR procedures described in the Wyoming PCR protocol.

December 2017. Twelve animals were sampled from the Targhee population in December 2017 using several protocols, in addition to the Wyoming Protocol, that deviated slightly from our other protocols. Nasal swab samples were collected from 12 animals and kept chilled in 3mL Amies media without charcoal, as previously described for up to 48 hours until received by the WGFD laboratory where they were analyzed according to the same procedures described for the “Wyoming Protocol”. These samples are recorded as “Port-A-Cul” samples in Table A below. A nasal swab and tonsil swab sample were collected from four animals and were frozen as soon as possible in separate TSB vial until received by the WGFD laboratory where they were each analyzed according to the same procedures described for the “Wyoming Protocol”. These samples are recorded as “TSB-Nasal” and “TSB” samples, respectively, in Table A below. Given the small number of animals these protocols were applied to, we did not define new protocols for these samples and our treatment of data from these samples is described in the “Evaluating Freedom from Infection” section below.

***Mycoplasma ovipneumoniae***

Wyoming*.* Samples from the nasal passages were obtained by inserting sterile polyester applicators (Puritan#25-806 1PD, Guilford, ME, USA) 8-12 cm into the nasal cavity and slowly rotating the shaft. The inoculated swab was then placed into transport media: Port-A-Cul^TM^ tubes (Becton Dickinson, Franklin Lakes, NJ, USA) or 3ml Amies media without charcoal in a 15 x 103mm culture tube (Triforest Enterprises, Irvine, CA, USA). Swabs were removed from the transport media as previously described and placed into 2ml of modified tryptone soy broth (TSB-1) in sterile 5ml round-bottom tubes (BD Falcon, Franklin Lakes, NJ, USA) and incubated at 37°C with 5% CO_2_ for 48 hours. DNA was extracted from 1 mL of the TSB-1 as described for the Wyoming PCR *Pasteurellaceae* protocol. DNA was analyzed using primers and PCR protocol published by McAuliffe [5], and optimized in the Wyoming Game and Fish Department Wildlife Health lab by modifying the initial denaturation for five minutes at 94°C, 32 denaturation cycles for 30 seconds each at 94°C, annealing at 57.5°C for 30 seconds, and extension at 72°C for 30 seconds. The final extension was at 72°C for 5 minutes. Samples were kept at 4°C until analyzed.

qPCR. A nasal swab was collected as described in the Wyoming PCR protocol and placed in a sterile cryovial without transport media and stored frozen. The samples were shipped to WADDL on dry ice and were tested for presence of *Mycoplasma ovipneumoniae* using quantitative PCR (qPCR) to aid in protocol development. This does not represent a commercially-available protocol.

TSB. A nasal swab was collected as described in the Wyoming PCR protocol and placed immediately into a vial of tryptic soy broth (TSB). Samples were frozen as soon as possible and shipped overnight on dry ice to Washington Animal Disease Diagnostic Laboratory (WADDL) for *Mycoplasma ovipneumoniae* PCR testing [5,6].

**Table A.**

|  | |  |  | |  | | **Diagnostic Protocols Used per Animal** | | | | | | |
| --- | --- | --- | --- | --- | --- | --- | --- | --- | --- | --- | --- | --- | --- |
| **Population** | | **Year** |  | | **Animals Sampled** | | Wyoming | Plated PCR | Plated Culture | Port-A-Cul | TSB | TSB-Nasal | Tissue |
| Clark's Fork | | 12.13 |  | | 1 | | 0 | 0 | 0 | 0 | 1 | 0 | 0 |
|  | | 12.13 |  | | 8 | | 1 | 0 | 0 | 0 | 1 | 0 | 0 |
|  | | 13.14 |  | | 3 | | 1 | 0 | 0 | 0 | 0 | 0 | 0 |
|  | | 14.15 |  | | 8 | | 1 | 0 | 0 | 0 | 0 | 0 | 0 |
|  | | **Total** |  | | **20** | |  |  |  |  |  |  |  |
| Dubois Badlands | | 13.14 |  | | 5 | | 1 | 0 | 0 | 0 | 0 | 0 | 0 |
|  | | 15.16 |  | | 1 | | 1 | 0 | 0 | 0 | 0 | 0 | 0 |
|  | | 15.6 |  | | 4 | | 1 | 0 | 0 | 0 | 1 | 0 | 0 |
|  | | **Total** |  | | **10** | |  |  |  |  |  |  |  |
| Fergus | | 14.15 |  | | 30 | | 0 | 0 | 0 | 0 | 1 | 0 | 0 |
|  | | 14.15 |  | | 15 | | 0 | 1 | 0 | 0 | 1 | 0 | 0 |
|  | | 14.15 |  | | 15 | | 0 | 1 | 1 | 0 | 1 | 0 | 0 |
|  | | 16.17 |  | | 30 | | 0 | 0 | 0 | 0 | 2 | 1 | 0 |
|  | | 16.17 |  | | 30 | | 0 | 0 | 0 | 0 | 2 | 0 | 0 |
|  | | **Total** |  | | **120** | |  |  |  |  |  |  |  |
| Galton | | 16.17 |  | | 31 | | 0 | 0 | 0 | 0 | 1 | 0 | 0 |
|  | | **Total** |  | | **31** | |  |  |  |  |  |  |  |
| Gibson Lake North | | 16.17 |  | | 12 | | 0 | 0 | 0 | 0 | 2 | 1 | 0 |
|  | | **Total** |  | | **12** | |  |  |  |  |  |  |  |
| Franc's Peak | | 12.13 |  | | 2 | | 1 | 0 | 0 | 0 | 1 | 0 | 0 |
|  | | 13.14 |  | | 12 | | 1 | 0 | 0 | 0 | 0 | 0 | 0 |
|  | | 14.15 |  | | 10 | | 1 | 0 | 0 | 0 | 0 | 0 | 0 |
|  | | 14.15 |  | | 3 | | 1 | 0 | 1 | 0 | 1 | 0 | 0 |
|  | | 15.16 |  | | 1 | | 1 | 0 | 0 | 0 | 0 | 0 | 0 |
|  | | 15.16 |  | | 1 | | 1 | 0 | 0 | 0 | 1 | 0 | 0 |
|  | | 16.17 |  | | **3** | | **1** | **0** | **0** | **0** | **0** | **0** | **0** |
|  | | **Total** |  | | **32** | |  |  |  |  |  |  |  |
| Highlands | | 15.16 |  | | 16 | | 0 | 0 | 0 | 0 | 2 | 0 | 0 |
|  | | **Total** |  | | **16** | |  |  |  |  |  |  |  |
| Hilgard | | 13.14 |  | | 29 | | 0 | 0 | 0 | 0 | 1 | 0 | 0 |
|  | | 14.15 |  | | 1 | | 0 | 0 | 0 | 0 | 0 | 0 | 1 |
|  | | 14.15 |  | | 1 | | 0 | 0 | 0 | 0 | 1 | 0 | 0 |
|  | | 14.15 |  | | 10 | | 0 | 0 | 0 | 0 | 2 | 0 | 0 |
|  | | 14.15 |  | | 20 | | 0 | 1 | 0 | 0 | 2 | 0 | 0 |
|  | | 14.15 |  | | 18 | | 0 | 1 | 1 | 0 | 2 | 0 | 0 |
|  | | 15.16 |  | | 3 | | 0 | 1 | 0 | 1 | 2 | 0 | 0 |
|  | | 15.16 |  | | 1 | | 0 | 1 | 1 | 1 | 2 | 0 | 0 |
|  | | 15.16 |  | | 1 | | 0 | 1 | 2 | 1 | 1 | 0 | 0 |
|  | | 15.16 |  | | 29 | | 0 | 1 | 2 | 1 | 2 | 0 | 0 |
|  | | 16.17 |  | | 30 | | 0 | 0 | 0 | 0 | 2 | 1 | 0 |
|  | | 16.17 |  | | 1 | | 0 | 0 | 0 | 0 | 2 | 0 | 0 |
|  | | **Total** |  | | **143** | |  |  |  |  |  |  |  |
| Jackson | | 12.13 |  | | 15 | | 1 | 0 | 0 | 0 | 0 | 0 | 0 |
|  | | 12.13 |  | | 1 | | 1 | 0 | 0 | 0 | 1 | 0 | 0 |
|  | | 13.14 |  | | 12 | | 1 | 0 | 0 | 0 | 0 | 0 | 0 |
|  | | 14.15 |  | | 12 | | 1 | 0 | 0 | 0 | 0 | 0 | 0 |
|  | | 15.16 |  | | 11 | | 1 | 0 | 0 | 0 | 0 | 0 | 0 |
|  | | 15.16 |  | | 16 | | 1 | 0 | 0 | 0 | 1 | 0 | 0 |
|  | | **Total** |  | | **67** | |  |  |  |  |  |  |  |
| Lost Creek | | 14.15 |  | | 13 | | 0 | 1 | 1 | 0 | 1 | 0 | 0 |
|  | | 15.16 |  | | 1 | | 0 | 0 | 2 | 0 | 2 | 0 | 0 |
|  | | 15.16 |  | | 5 | | 0 | 1 | 2 | 0 | 2 | 0 | 0 |
|  | | 16.17 |  | | 23 | | 0 | 0 | 0 | 0 | 2 | 1 | 0 |
|  | | 16.17 |  | | 1 | | 0 | 0 | 0 | 0 | 1 | 0 | 0 |
|  | | **Total** |  | | **43** | |  |  |  |  |  |  |  |
| Middle Missouri Breaks | | 15.16 |  | | 19 | | 0 | 0 | 0 | 0 | 2 | 0 | 0 |
|  | 16.17 | | |  | | 20 | 0 | 0 | 0 | 0 | 2 | 1 | 0 |
|  | | **Total** |  | | **39** | |  |  |  |  |  |  |  |
| Perma-Paradise | | 14.15 |  | | 2 | | 0 | 0 | 0 | 0 | 1 | 0 | 0 |
|  | | 14.15 |  | | 13 | | 0 | 1 | 0 | 0 | 1 | 0 | 0 |
|  | | 14.15 |  | | 15 | | 0 | 1 | 1 | 0 | 1 | 0 | 0 |
|  | | 16.17 |  | | 29 | | 0 | 0 | 0 | 0 | 2 | 1 | 0 |
|  | | 16.17 |  | | 1 | | 0 | 0 | 0 | 0 | 1 | 1 | 0 |
|  | | **Total** |  | | **30** | |  |  |  |  |  |  |  |
| Petty Creek | | 15.16 |  | | 1 | | 0 | 1 | 2 | 1 | 0 | 0 | 0 |
|  | | 15.16 |  | | 16 | | 0 | 1 | 2 | 1 | 2 | 0 | 0 |
|  | | **Total** |  | | **17** | |  |  |  |  |  |  |  |
| Stillwater | | 14.15 |  | | 16 | | 0 | 1 | 1 | 0 | 1 | 0 | 0 |
|  | | 15.16 |  | | 3 | | 0 | 1 | 0 | 0 | 2 | 0 | 0 |
|  | | 16.17 |  | | 11 | | 0 | 0 | 0 | 0 | 2 | 1 | 0 |
|  | | **Total** |  | | **30** | |  |  |  |  |  |  |  |
| Sun Canyon | | 14.15 |  | | 2 | | 0 | 0 | 0 | 0 | 1 | 0 | 0 |
|  | | 14.15 |  | | 5 | | 0 | 1 | 0 | 0 | 1 | 0 | 0 |
|  | | 14.15 |  | | 16 | | 0 | 1 | 1 | 0 | 1 | 0 | 0 |
|  | | 15.16 |  | | 7 | | 0 | 1 | 2 | 0 | 2 | 0 | 0 |
|  | | 16.17 |  | | 25 | | 0 | 0 | 0 | 0 | 2 | 1 | 0 |
|  | | **Total** |  | | **55** | |  |  |  |  |  |  |  |
| Targhee^1^ | | 16.17 |  | | 6 | | 6 | 0 | 0 | 0 | 0 | 0 | 0 |
|  | | 17.18 |  | | 8 | | 8 | 0 | 0 | 8 | 0 | 0 | 0 |
|  | | 17.18 |  | | 4 | | 0 | 0 | 0 | 4 | 4 | 4 | 0 |
|  | | **Total** |  | | **18** | |  |  |  |  |  |  |  |
| Trout Peak | | 12.13 |  | | 4 | | 1 | 0 | 0 | 0 | 1 | 0 | 0 |
|  | | 13.14 |  | | 9 | | 1 | 0 | 0 | 0 | 0 | 0 | 0 |
|  | | 14.15 |  | | 1 | | 0 | 0 | 0 | 0 | 2 | 0 | 0 |
|  | | 14.15 |  | | 2 | | 1 | 0 | 0 | 0 | 0 | 0 | 0 |
|  | | 15.16 |  | | 1 | | 1 | 0 | 0 | 0 | 0 | 0 | 0 |
|  | | 15.16 |  | | 7 | | 1 | 0 | 0 | 0 | 1 | 0 | 0 |
|  | | **Total** |  | | **24** | |  |  |  |  |  |  |  |
| Wapiti Ridge | | 12.13 |  | | 16 | | 1 | 0 | 0 | 0 | 1 | 0 | 0 |
|  | | 13.14 |  | | 13 | | 1 | 0 | 0 | 0 | 0 | 0 | 0 |
|  | | 14.15 |  | | 3 | | 1 | 0 | 0 | 0 | 0 | 0 | 0 |
|  | | 14.15 |  | | 7 | | 1 | 0 | 1 | 0 | 1 | 0 | 0 |
|  | | 15.16 |  | | 10 | | 1 | 0 | 0 | 0 | 0 | 0 | 0 |
|  | | 15.16 |  | | 10 | | 1 | 0 | 0 | 0 | 1 | 0 | 0 |
|  | | 16.17 |  | | 11 | | 1 | 0 | 0 | 0 | 0 | 0 | 0 |
|  | | **Total** |  | | **70** | |  |  |  |  |  |  |  |
| Yount’s Peak | | 16.17 |  | | 6 | | 1 | 0 | 0 | 0 | 0 | 0 | 0 |
|  | | **Total** |  | | **6** | |  |  |  |  |  |  |  |
| Whiskey Mountain | | 13.14 |  | | 3 | | 1 | 0 | 0 | 0 | 0 | 0 | 0 |
|  | | 14.15 |  | | 12 | | 1 | 0 | 0 | 0 | 0 | 0 | 0 |
|  | | 15.16 |  | | 10 | | 1 | 0 | 0 | 0 | 0 | 0 | 0 |
|  | | 15.16 |  | | 12 | | 1 | 0 | 0 | 0 | 1 | 0 | 0 |
|  | | **Total** |  | | **37** | |  |  |  |  |  |  |  |
| Upper Yellowstone Pre-2013 | | 11.12 |  | | 6 | | 0 | 0 | 0 | 1 | 0 | 0 | 0 |
|  | | 12.13 |  | | 11 | | 0 | 0 | 0 | 0 | 1 | 0 | 0 |
|  | | **Total** |  | | **17** | |  |  |  |  |  |  |  |
| Upper Yellowstone Post-2014 | | 14.15 |  | | 15 | | 0 | 0 | 0 | 0 | 0 | 0 | 1 |
|  | | **Total** |  | | **15** | |  |  |  |  |  |  |  |

**^1.^** Samples listed under the Port-Cul, TSB, and TSB-Nasal protocols for the Targhee population were collected following different procedures as previously described. See previous section for protocol descriptions and subsequent section for description of how these differences were addressed in analysis.

**Table B.**

|  |  |  |  | **Diagnostic Protocols** | | | |
| --- | --- | --- | --- | --- | --- | --- | --- |
| **Population** | **Year** |  | **Animals Sampled** | Wyoming | TSB | qPCR | Tissue |
| Clark's Fork | 12.13 |  | 6 | 0 | 1 | 0 | 0 |
|  | 12.13 |  | 2 | 1 | 0 | 0 | 0 |
|  | 12.13 |  | 1 | 1 | 1 | 0 | 0 |
|  | 13.14 |  | 3 | 1 | 0 | 0 | 0 |
|  | 14.15 |  | 8 | 1 | 0 | 0 | 0 |
|  | **Total** |  | **20** |  |  |  |  |
| Dubois Badlands | 13.14 |  | 5 | 1 | 0 | 0 | 0 |
|  | 15.16 |  | 5 | 1 | 1 | 0 | 0 |
|  | **Total** |  | **10** |  |  |  |  |
| Galton | 16.17 |  | 31 | 0 | 1 | 0 | 0 |
|  | **Total** |  | **31** |  |  |  |  |
| Gibson Lake North | 16.17 |  | 12 | 0 | 1 | 0 | 0 |
|  | **Total** |  | **12** |  |  |  |  |
| Fergus | 14.15 |  | 30 | 0 | 1 | 0 | 0 |
|  | 14.15 |  | 29 | 0 | 1 | 1 | 0 |
|  | 16.17 |  | 60 | 0 | 1 | 0 | 0 |
|  | **Total** |  | **119** |  |  |  |  |
| Franc's Peak | 12.13 |  | 2 | 1 | 1 | 0 | 0 |
|  | 13.14 |  | 12 | 1 | 0 | 0 | 0 |
|  | 14.15 |  | 13 | 1 | 0 | 0 | 0 |
|  | 15.16 |  | 2 | 1 | 1 | 0 | 0 |
|  | 16.17 |  | 3 | 1 | 0 | 0 | 0 |
|  | **Total** |  | **32** |  |  |  |  |
| Highlands | 15.16 |  | 16 | 0 | 2 | 0 | 0 |
|  | **Total** |  | **16** |  |  |  |  |
| Hilgard | 13.14 |  | 29 | 0 | 1 | 0 | 0 |
|  | 13.14 |  | 1 | 0 | 0 | 0 | 1 |
|  | 13.14 |  | 50 | 0 | 1 | 0 | 0 |
|  | 15.16 |  | 35 | 0 | 2 | 0 | 0 |
|  | 16.17 |  | 31 | 0 | 1 | 0 | 0 |
|  | **Total** |  | **145** |  |  |  |  |
| Jackson | 12.13 |  | 1 | 0 | 1 | 0 | 0 |
|  | 12.13 |  | 13 | 1 | 0 | 0 | 0 |
|  | 13.14 |  | 13 | 1 | 0 | 0 | 0 |
|  | 14.15 |  | 12 | 1 | 0 | 0 | 0 |
|  | 14.15 |  | 7 | 1 | 0 | 0 | 0 |
|  | 15.16 |  | 16 | 1 | 1 | 0 | 0 |
|  | **Total** |  | **62** |  |  |  |  |
| Lost Creek | 14.15 |  | 6 | 0 | 1 | 0 | 0 |
|  | 14.15 |  | 7 | 0 | 1 | 1 | 0 |
|  | 15.16 |  | 6 | 0 | 2 | 0 | 0 |
|  | 16.17 |  | 24 | 0 | 1 | 0 | 0 |
|  | **Total** |  | **43** |  |  |  |  |
| Middle Missouri Breaks | 15.16 |  | 19 | 0 | 2 | 0 | 0 |
|  | 16.17 |  | 20 | 0 | 1 | 0 | 0 |
|  | **Total** |  | **39** |  |  |  |  |
| Perma-Paradise | 14.15 |  | 30 | 0 | 1 | 1 | 0 |
|  | 16.17 |  | **29** | 0 | 1 | 0 | 0 |
|  | **Total** |  | **59** |  |  |  |  |
| Petty Creek | 15.16 |  | 16 | 0 | 2 | 0 | 0 |
|  | **Total** |  | **16** |  |  |  |  |
| Stillwater | 14.15 |  | 9 | 0 | 1 | 0 | 0 |
|  | 14.15 |  | 7 | 0 | 1 | 1 | 0 |
|  | 15.16 |  | 3 | 0 | 2 | 0 | 0 |
|  | 16.17 |  | 11 | 0 | 1 | 0 | 0 |
|  | **Total** |  | **30** |  |  |  |  |
| Sun Canyon | 14.15 |  | 8 | 0 | 1 | 0 | 0 |
|  | 14.15 |  | 15 | 0 | 1 | 1 | 0 |
|  | 15.16 |  | 7 | 0 | 2 | 0 | 0 |
|  | 16.17 |  | 25 | 0 | 1 | 0 | 0 |
|  | **Total** |  | **55** |  |  |  |  |
| Targhee | 16.17 |  | 6 | 1 | 0 | 0 | 0 |
|  | 17.18 |  | 12 | 1 | 0 | 0 | 0 |
|  | **Total** |  | **18** |  |  |  |  |
| Trout Peak | 12.13 |  | 4 | 1 | 1 | 0 | 0 |
|  | 13.14 |  | 9 | 1 | 0 | 0 | 0 |
|  | 14.15 |  | 2 | 0 | 1 | 0 | 0 |
|  | 14.15 |  | 2 | 1 | 0 | 0 | 0 |
|  | 15.16 |  | 8 | 1 | 1 | 0 | 0 |
|  | **Total** |  | **25** |  |  |  |  |
| Wapiti Ridge | 12.13 |  | 16 | 1 | 1 | 0 | 0 |
|  | 13.14 |  | 13 | 1 | 0 | 0 | 0 |
|  | 14.15 |  | 10 | 1 | 0 | 0 | 0 |
|  | 15.16 |  | 3 | 1 | 0 | 0 | 0 |
|  | 15.16 |  | 14 | 1 | 1 | 0 | 0 |
|  | 16.17 |  | 11 | 1 | 0 | 0 | 0 |
|  | **Total** |  | **67** |  |  |  |  |
| Yount’s Peak | 16.17 |  | 6 | 1 | 0 | 0 | 0 |
|  | **Total** |  | **6** |  |  |  |  |
| Whiskey Mountain | 13.14 |  | 3 | 1 | 0 | 0 | 0 |
|  | 14.15 |  | 12 | 1 | 0 | 0 | 0 |
|  | 15.16 |  | 8 | 1 | 0 | 0 | 0 |
|  | 15.16 |  | 12 | 1 | 1 | 0 | 0 |
|  | **Total** |  | **35** |  |  |  |  |
| Upper Yellowstone Pre-2013 | 11.12 |  | 6 | 0 | 1 | 0 | 0 |
|  | 12.13 |  | 11 | 0 | 1 | 0 | 0 |
|  | **Total** |  | **17** |  |  |  |  |
| Upper Yellowstone Post-2014 | 14.15 |  | 15 | 0 | 0 | 0 | 1 |
|  | **Total** |  | **15** |  |  |  |  |


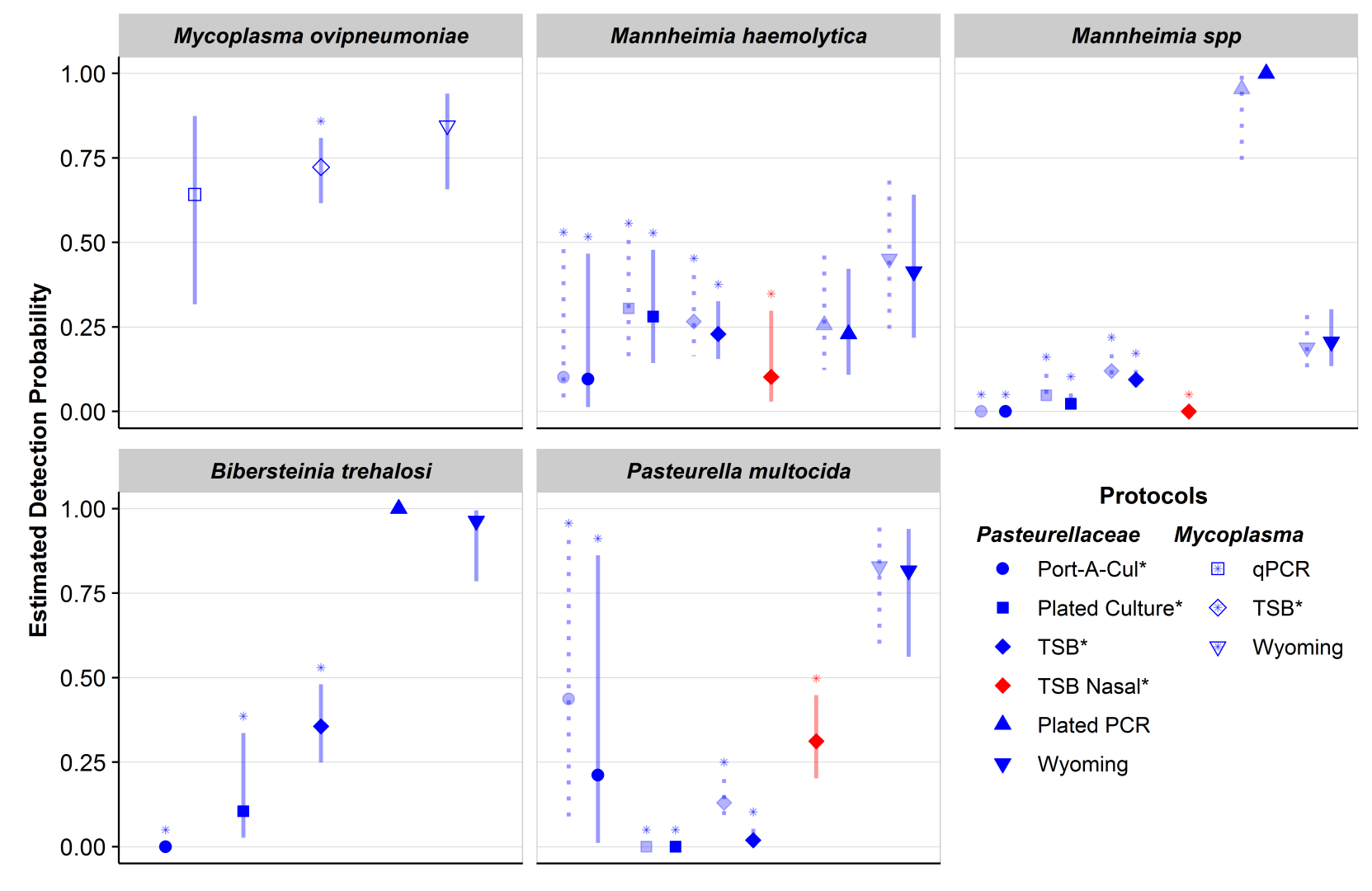


**Fig A.**

**Table C.**

| **Pathogen** | **Protocol** | **Distribution Parameters-Original** | | **Distribution Parameters-Updated** | |
| --- | --- | --- | --- | --- | --- |
|  |  | *Logit-Normal*  (μ,σ^2^) | *Beta*  (α, β) | *Logit-Normal*  (μ,σ^2^) | *Beta*  (α, β) |
| *Mannheimia haemolytica* | |  |  |  |  |
| Plated PCR | | -1.07, 0.46 | 6.63, 18.36 | -1.21,0.46 | **6.51, 20.74** |
| Plated Culture | | -0.83, 0.44 | 8, 17.65 | -0.94, 0.43 | **7.79, 19.16** |
| Port-A-Cul | | -2.18, 1.07 | 1.22, 7.34 | -2.24, 1.07 | **1.21, 7.68** |
| TSB | | -1.02, 0.32 | 14.01, 37.82 | -1.21, 0.24 | **21.60, 71.51** |
| TSB-Nasal | | -- | -- | -2.18, 0.67 | **2.72, 20.22** |
| Wyoming | | -0.19, 0.49 | 8.08, 9.71 | -0.35, 0.12 | **129.40, 182.96** |
| *Mannheimia spp.* | |  |  |  |  |
| Plated PCR**^2^** | | 3.01, 1.02 | **16.11, 1.22** | -- | -- |
| Plated Culture | | -2.99, 0.46 | 5.13, 93.05 | -3.75,0.45 | **5.18, 199.67** |
| Port-A-Cul^3^ | | -- | -- | -- | -- |
| TSB | | -2, 0.2 | 27.96, 202.63 | -2.27,0.15 | **49.46, 472.33** |
| TSB-Nasal | | -- | -- | -- | -- |
| Wyoming | | -1.46, 0.28 | 16.66, 69.99 | -1.35,0.04 | **682.18, 2628.04** |
| *Bibersteinia trehalosi* | |  |  |  |  |
| Plated PCR | | -- | **23.28, 41.64** | -- | -- |
| Plated Culture | | -2.15, 0.75 | **2.26, 15.79** | -- | -- |
| Port-A-Cul^3^ | | -- | -- | -- | -- |
| TSB | | -0.59, 0.26 | **23.28, 41.64** | -- | -- |
| TSB-Nasal | | -- | -- | -- | -- |
| Wyoming | | 3.29, 1.02 | **20.42, 1.2** | -- | -- |
| *Pasteurella multocida* | |  |  |  |  |
| Plated Culture^3^ | | --- | -- | -- | -- |
| Port-A-Cul | | -0.25, 1.29 | 1.48, 1.77 | -1.31,1.61 | **0.81, 1.91** |
| TSB | | -1.9, 0.26 | 16.84, 110.23 | -3.94,0.54 | **3.74, 168.49** |
| TSB-Nasal | | -- | -- | -0.79,0.30 | **16.93, 36.72** |
| Wyoming | | 1.58, 0.63 | 14.48, 3.36 | 1.50,0.10 | **605.17, 135.93** |
| *Mycoplasma ovipneumoniae* | |  |  |  |  |
| qPCR | | 0.59, 0.69 | **6.28, 3.69** | -- | -- |
| TSB | | 0.98, 0.25 | **60.18, 22.91** | -- | -- |
| Wyoming PCR | | 1.85, 0.53 | **25, 4.36** | -- | -- |

^1.^ There were no estimates of detection probability for lung tissue samples and a uniform beta distribution was used to reflect the lack of information.

^2^ The standard error of detection probability for this protocol could not be estimated in the updated dataset because detection probability was estimated at 1.

^3.^ Detection probability could not be directly estimated because these protocols never detected the respective pathogens. Detection probability in these instances was set at zero.

**
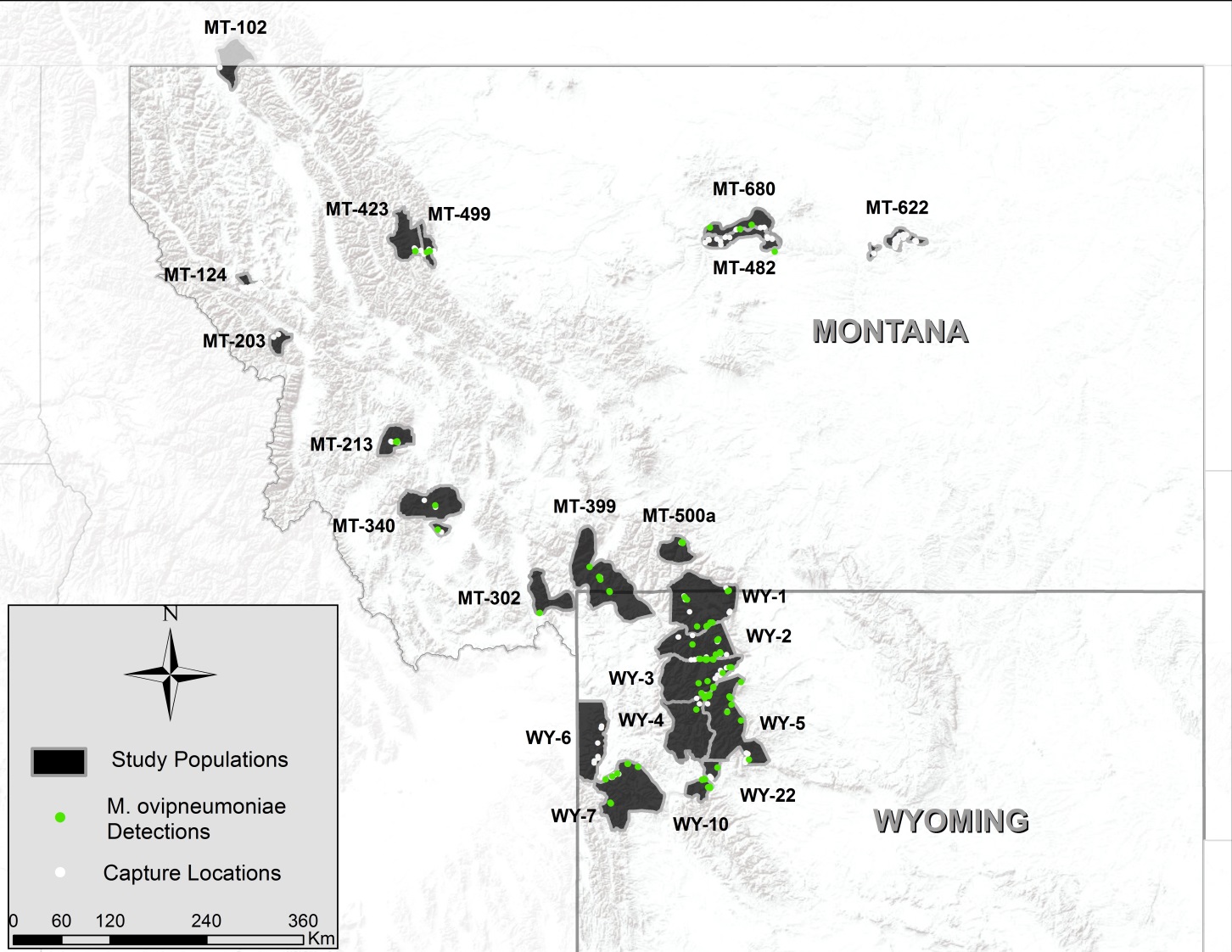
**

**Fig B.**.

**
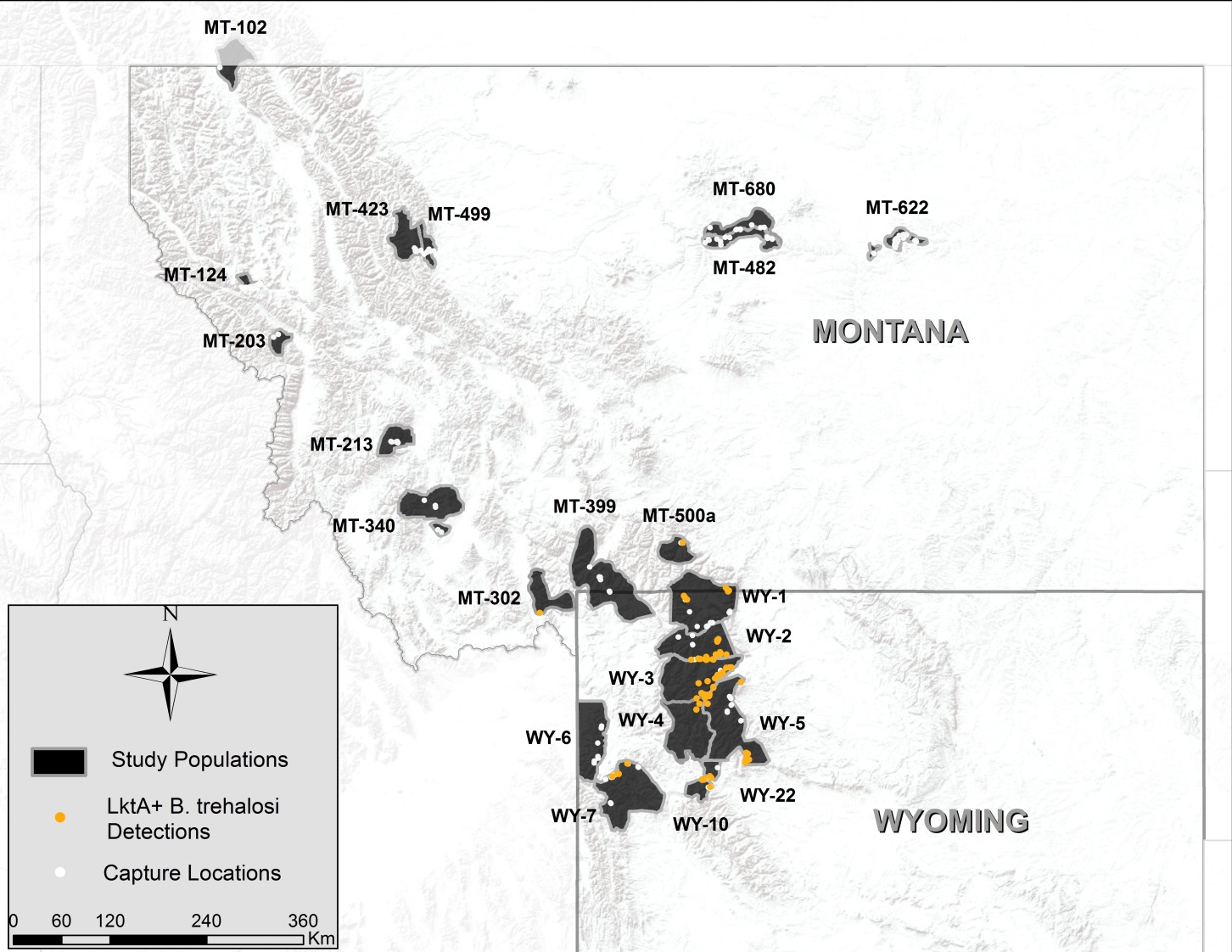
**

**Fig C.**

**
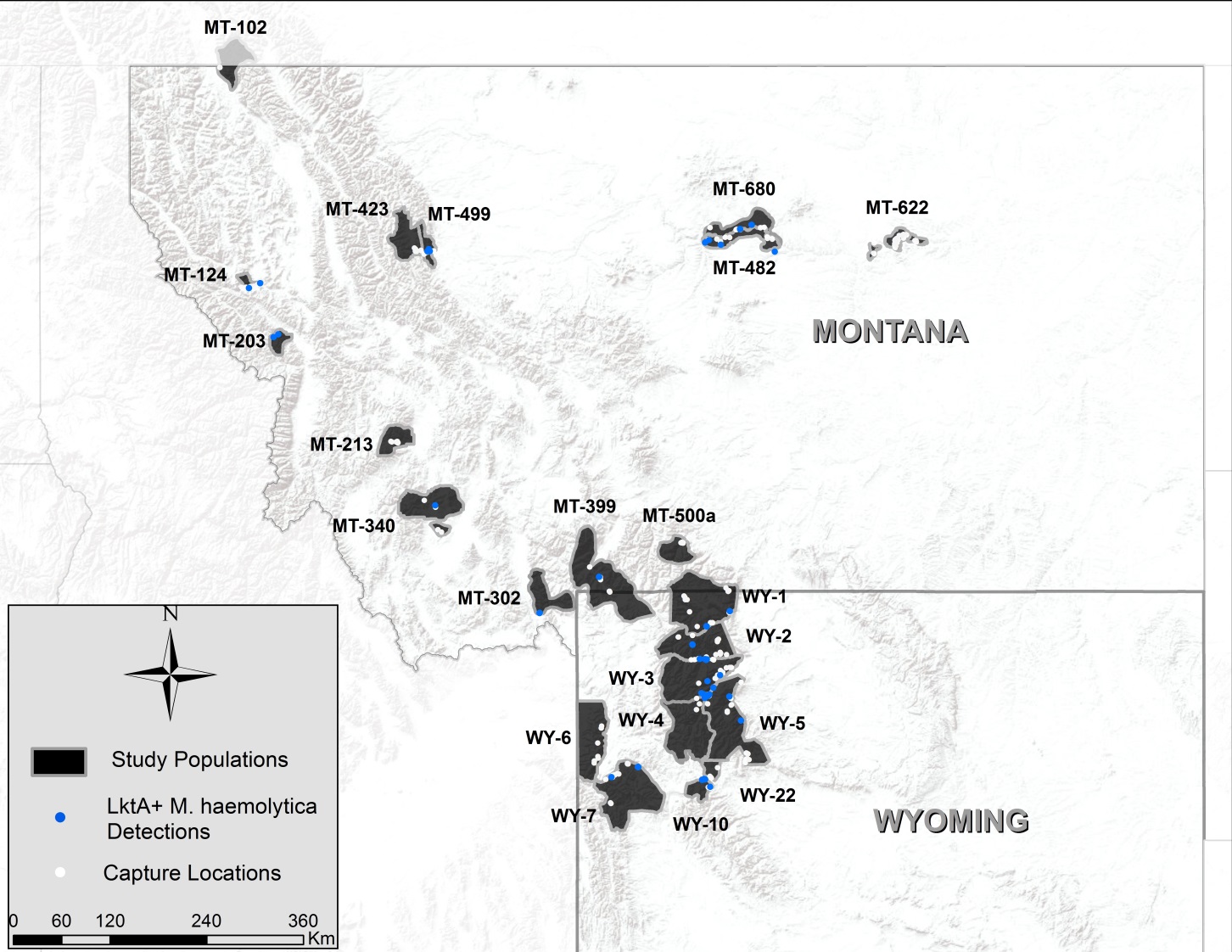
**

**Fig D.**

**
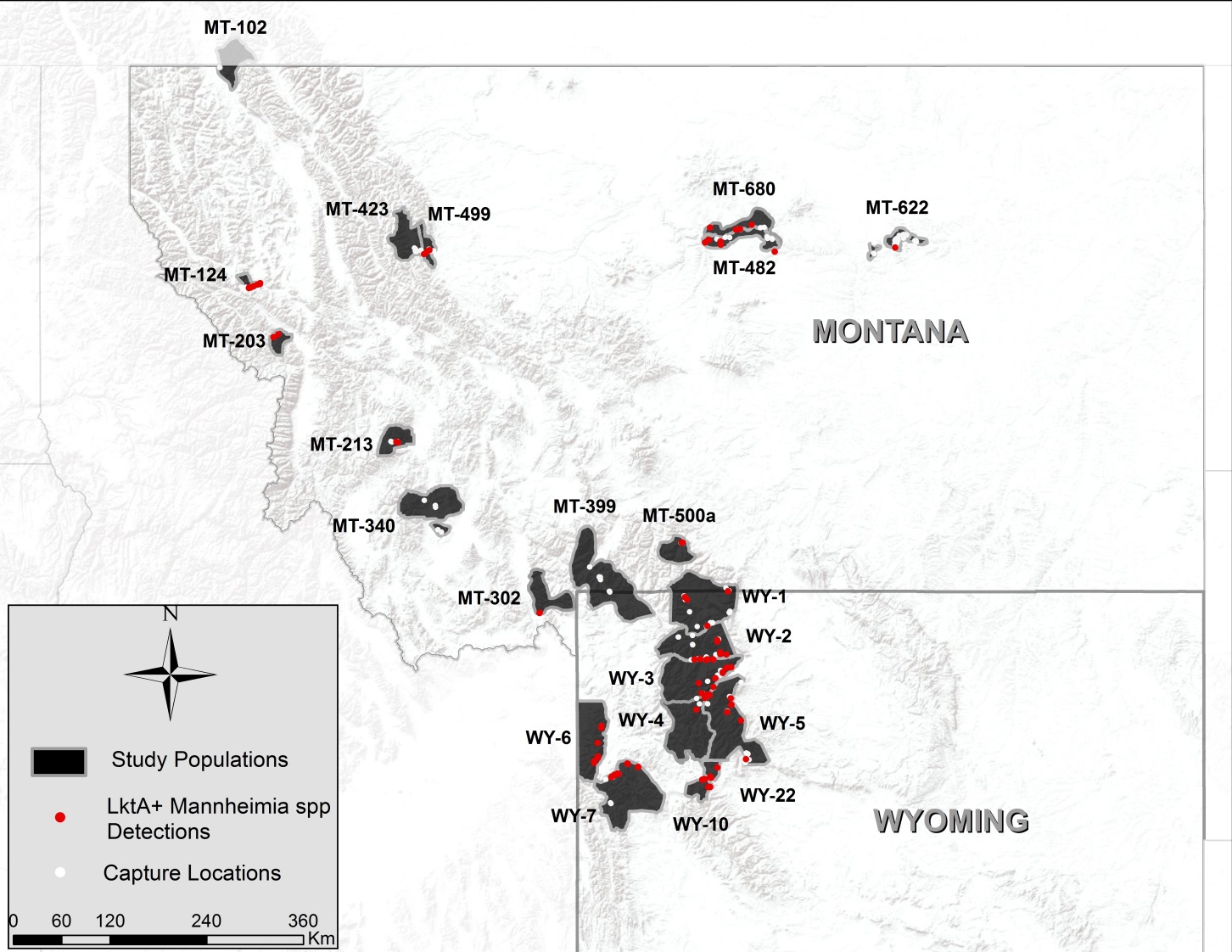
**

**Fig E**

**
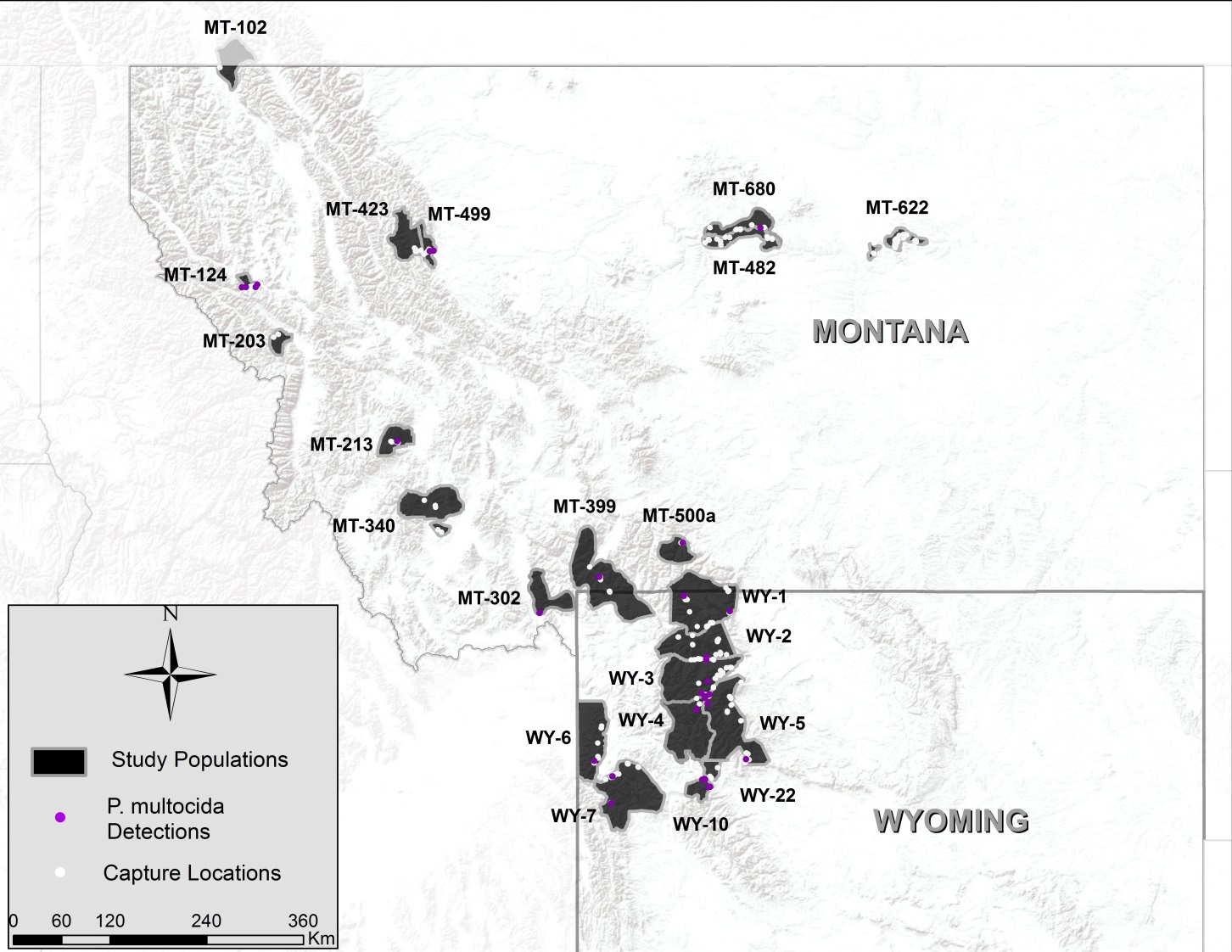
**

**Fig F.**

**
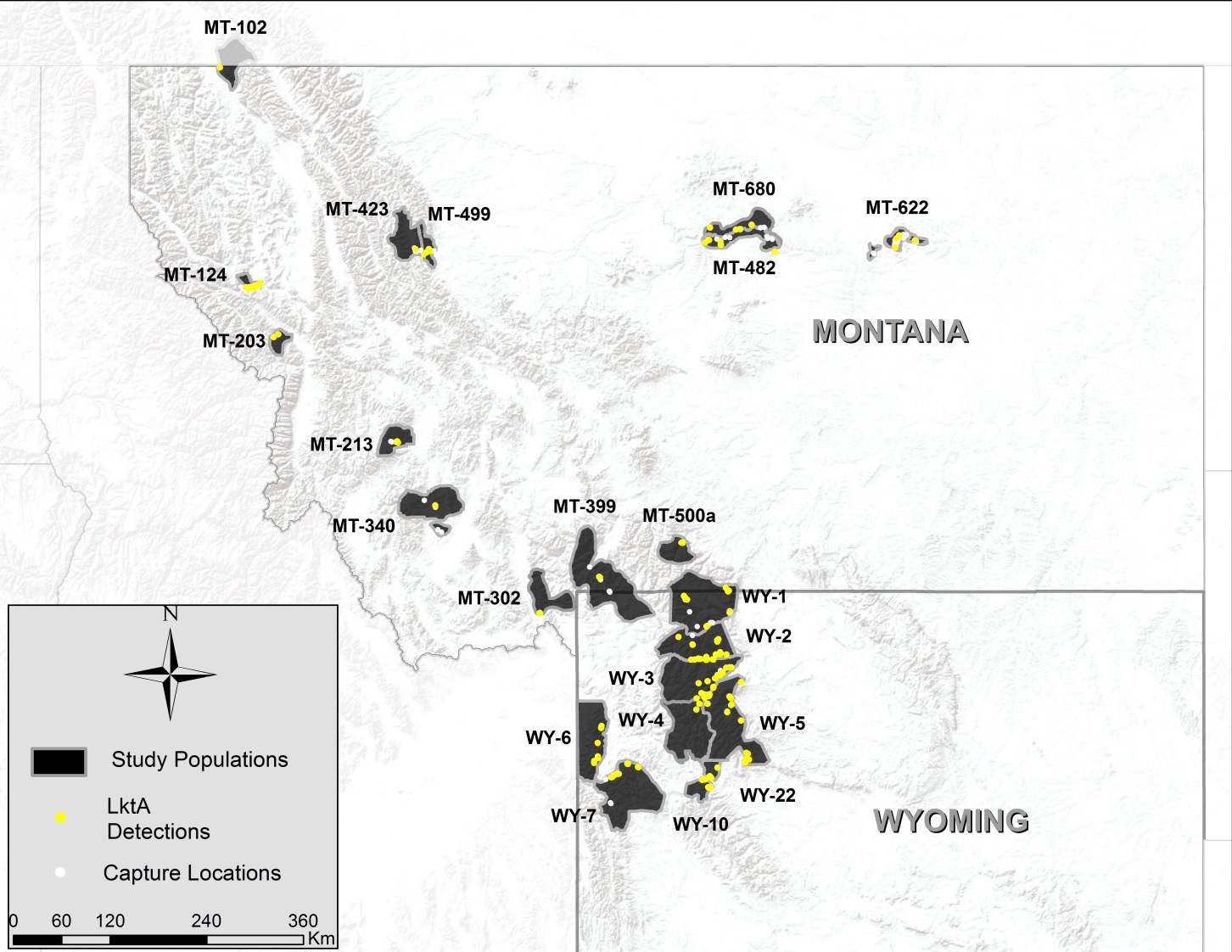
**

**Fig G.**

**Evaluating Freedom from Infection**

The probability of pathogen presence in a population despite no detections was estimated using a Bayesian hierarchical approach that incorporated uncertainty in the probabilities of detection to model prevalence and pathogen presence. The presence of pathogen *i* in population *h* was considered a Bernoulli random variable:

$$\mathrm{Presence}_{ih}= \left\{ \begin{aligned} 1, with probability \tau\\ 0, with probability 1-\tau\end{aligned} \right.$$

where$\tau=0.5$ for our analysis. Conditional on the presence of pathogen *i* in population *h*, the prevalence of the pathogen,$\pi_{ih}$, was modeled using a beta distribution:${\pi_{ih} \sim Beta\left( \alpha_{i}^{\pi},\beta_{i}^{\pi} \right) when pathogen is present \atop\pi_{ih}=0 when pathogen is absent,}$

where $\alpha_{i}=\beta_{i}=1$ (a uniform prior) was used for our analysis. This construction is a mixture distribution for prevalence [7,8] that facilitates the estimation of true prevalence by allowing $\pi_{ih}=0$, and ultimately allows an estimation for the probability of pathogen presence in a population. Conditional on the prevalence of pathogen *i* in population *h* and the probability of detection for pathogen *i* using protocol *j*, $p_{ij}$, the probability of a negative test result is:

$$\Pr\left( T=0 | \pi_{ih},p_{ij} \right)= \pi_{ih}\left( 1-p_{ij} \right)+ 1- \pi_{ih},$$

where $p_{ij} \sim\mathrm{Beta}\left( \alpha_{i}^{p},\beta_{i}^{p} \right)$, with $\alpha_{i}^{p},\beta_{i}^{p}$ set for each pathogen. The first term in the above equation represents the probability that an individual carries the pathogen, but the protocol failed to detect it, whereas the latter term represents the probability that the pathogen was absent.

When multiple samples per protocol and/or multiple protocols were used on an individual, the probability that every test is negative was:

$$\Pr\left( T=0 | \pi_{ih},p_{ij} \right)= \pi_{ih}\left( 1-p_{i}^{*} \right)+ 1- \pi_{ih},$$

where $p_{i}^{*}$is the probability of at least one detection of pathogen *i*, i.e. the probability that all results are negative is the complement of the probability of at least one detection. For multiple samples and multiple protocols (assuming independence), the probability of at least one detection was:

$$p_{i}^{*}= 1- \prod_{j=1}^{J} \left( 1-p_{ij} \right)^{n_{ij}},$$

where $n_{ij}$ is the number of samples for protocol *j* (for *j* = 1, …, J total protocols) and pathogen *i* in an individual. For example, if individual A was tested for the presence of pathogen *i* using protocol 1 (2 swabs) and protocol 2 (1 swab), the probability of all tests being negative is:

$$\Pr\left( T=0 | \pi_{ih},p_{ij} \right)= \pi_{ih}\left( 1-p_{i}^{*} \right)+ 1- \pi_{ih},$$

$$\Pr\left( T=0 | \pi_{ih},p_{ij} \right)= \pi_{ih}\left( 1-\left( 1- \prod_{j=1}^{J} \left( 1-p_{ij} \right)^{n_{ij}} \right) \right)+ 1- \pi_{ih},$$

$$\Pr\left( T=0 | \pi_{ih},p_{ij} \right)= \pi_{ih}\left( 1-\left( 1- \left( 1-p_{i1} \right)^{2}\left( 1-p_{i2} \right) \right) \right)+ 1- \pi_{ih},$$

$$\Pr\left( T=0 | \pi_{ih},p_{ij} \right)= \pi_{ih}\left( \left( 1-p_{i1} \right)^{2}\left( 1-p_{i2} \right) \right)+ 1- \pi_{ih}.$$

For multiple individuals, k = 1, …, K, subject to the same combination of multiple samples and/or protocols, and assuming binomial sampling, the probability that all tests are negative is:

$$\Pr\left( \sum_{k=1}^{K} T_{k}=0 | \pi_{ih},p_{ij} \right)= \left[ \pi_{ih}\left( 1-\left( 1- \prod_{j=1}^{J} \left( 1-p_{ij} \right)^{n_{ij}} \right) \right)+ 1- \pi_{ih} \right]^{K}$$

Where pathogen sampling occurred over multiple years and a pathogen was never detected, it was assumed that the pathogen status (present, absent) was identical through the entire duration of sampling, though we allowed prevalence to change among years.

The model was estimated using the runjags interface [9] to the JAGS (Just Another Gibbs Sampler) program for Bayesian analysis [10]. Though not strictly necessary, we coded the model using the “ones trick” [11] to explicitly write out the probabilities of no detections for a wide variety of combinations of sampling and protocols. We ran four separate chains for 100,000 iterations, discarding the first 10,000 as burn-in. Convergence was assessed through a graphical inspection of chains [12], and inference based on the combined 360,000 iterations.

We could not reliably estimate detection probability for one pathogen-protocol combination (*B. trehalosi-*Plated PCR) because the pathogen was only detected in two animals where the protocol was used. Both detections were from the Plated PCR protocol suggesting detection probability is higher than for the other protocols used on the same set of animals. When estimating probability that *B. trehalosi* was present in the populations where it was not detected and the Plated PCR protocol was employed, we used the detection probability parameters for the TSB protocol (the protocol with the highest estimable detection probability that was also used on the same set of individuals) to represent the Plated PCR protocol, and when reporting the probability of presence for *B. trehalosi* we denoted that the true probability was lower than reported. Detection probability for several pathogen-protocol combinations could not be estimated because the protocol never detected the pathogen (*Mannheimia spp.*-Port-A-Cul, *Mannheimia spp.-*TSB-Nasal, *B. trehalosi-*Port-A-Cul, *B. trehalosi-*TSB-Nasal, *P. multocida*-Plated Culture), suggesting lower detection probability than for protocols that did detect these pathogens. For populations where these protocols were applied and the specified pathogen was not detected, we estimated probability of pathogen presence assuming detection probability for these pathogen-protocol combinations was zero, but denoted that the true probability of presence was lower than estimated, since detection probability for these protocols was likely greater than zero. We had no information regarding detection probabilities of any diagnostic test (e.g., culture or PCR) applied to lung tissues and we reflected this by using a uniform beta distribution (α=1, β=1) to define detection probability when tissue samples were used to assess pathogen presence. This is only applicable to the Upper Yellowstone Complex in 2014/2015, where *B. trehalosi* and *Mannheimia spp.* were not detected. We used detection probability parameters derived from our update of Butler et al. 2017 [13] as prior distributions in the model wherever possible, however were forced to rely on the original parameters from Butler et al. 2017 for one protocol (*Mannheimia* *spp.-*Plated PCR) because the updated detection probability for this protocol was estimated at 1 and the standard error could not be estimated (Fig A). For nasal and tonsil swabs that were collected from four animals in the Targhee population and frozen in TSB before analysis at WGFD, we used the detection probability parameter of the TSB-Nasal and TSB *Pasteurellaceae* protocols as a conservative prior, given the additional PCR tests that WGFD conducts. For nasal swabs that were collected from 12 animals in the Targhee population and chilled in Amies media without charcoal, we used a uniform beta distribution (α=1, β=1) as a prior to reflect the lack of information about detection probability. For *Pasteurellaceae* that were undetected in the Targhee population we denoted the true probability of presence was lower than estimated given the conservative priors that were used. All the approaches that we used to address issues related to unreliable detection probability estimates erred on the side of reducing type II errors (i.e. falsely concluding an undetected pathogen was truly absent).

**References**

1. Quinn PJ, Markey BK, Leonard FC, FitzPatrick ES, Fanning S, Hartigan P. Veterinary Microbiology and Microbial Disease. John Wiley & Sons; 2011.

2. Dassanayake RP, Shanthalingam S, Subramaniam R, Herndon CN, Bavananthasivam J, Haldorson GJ, et al. Role of Bibersteinia trehalosi, respiratory syncytial virus, and parainfluenza-3 virus in bighorn sheep pneumonia. Vet Microbiol. 2013;162: 166–172. doi:10.1016/j.vetmic.2012.08.029

3. Shanthalingam S, Goldy A, Bavananthasivam J, Subramaniam R, Batra SA, Kugadas A, et al. PCR assay detects Mannheimia haemolytica in culture-negative pneumonic lung tissues of bighorn sheep (Ovis canadensis) from outbreaks in the western USA, 2009-2010. J Wildl Dis. 2014;50: 1–10. doi:10.7589/2012-09-225

4. Angen O, Thomsen J, Larsen LE, Larsen J, Kokotovic B, Heegaard PMH, et al. Respiratory disease in calves: Microbiological investigations on trans-tracheally aspirated bronchoalveolar fluid and acute phase protein response. Vet Microbiol. 2009;137: 165–171. doi:10.1016/j.vetmic.2008.12.024

5. McAuliffe L, Ellis RJ, Ayling RD, Nicholas RAJ. Differentiation of Mycoplasma species by 16S ribosomal DNA PCR and denaturing gradient gel electrophoresis fingerprinting. J Clin Microbiol. 2003;41: 4844–4847.

6. Lawrence PK, Shanthalingam S, Dassanayake RP, Subramaniam R, Herndon CN, Knowles DP, et al. Transmission of Mannheimia haemolytica from domestic sheep (Ovies aries) to bighorn sheep (Ovis canadensis): Unequivocol demonstration with green fluorescent protein-tagged organisms. J Wildl Dis. 2010;46: 706–717.

7. Hanson TE, Johnson WO, Gardner IA, Georgiadis MP. Determining the infection status of a herd. J Agric Biol Environ Stat. 2003;8: 469–485. Available: http://www.jstor.org.proxybz.lib.montana.edu/stable/1400669

8. Branscum AJ, Gardner IA, Johnson WO. Bayesian modeling of animal- and herd-level prevalences. Prev Vet Med. 2004;66: 101–112. doi:10.1016/j.prevetmed.2004.09.009

9. Denwood MJ. runjags: An R package providing interface utilities, model templates, parallel computing methods and additional distributions for MCMC models in JAGS. J Stat Softw. 2016;71. doi:10.18637/jss.v071.i09

10. Plummer M. JAGS: A program for analysis of Bayesian graphical models using Gibbs sampling. Proceedings of the 3rd international workshop on distributed statistical computing. Vienna, Austria; 2003. p. 125.

11. Ntzoufras I. Bayesian modeling using WinBUGS [Internet]. Hoboken, New Jersey, United States: Wiley; 2009. Available: http://ebookcentral.proquest.com/lib/montana/detail.action?docID=427808

12. Gelman A, Carlin JB, Stern HS, Dunson DB, Vehtari A, Rubin DB. Bayesian Data Analysis, Third Edition [Internet]. Philadelphia, PA, UNITED STATES: CRC Press; 2013. Available: http://ebookcentral.proquest.com/lib/montana/detail.action?docID=1438153

13. Butler CJ, Edwards WH, Jennings-Gaines JE, Killion HJ, Wood ME, McWhirter DE, et al. Assessing respiratory pathogen communities in bighorn sheep populations: Sampling realities, challenges, and improvements. PLoS ONE. 2017;12: e0180689. doi:10.1371/journal.pone.0180689
